# Numerical Simulation of Rheological Models for Complex Fluids Using Hierarchical Grids

**DOI:** 10.3390/polym14224958

**Published:** 2022-11-16

**Authors:** Hugo A. Castillo-Sánchez, Leandro F. de Souza, Antonio Castelo

**Affiliations:** Institute of Mathematics and Computer Sciences, University of São Paulo, Av. Trab. São Carlense, 400, Centro, São Carlos 13566-590, SP, Brazil

**Keywords:** CFD, rheology, complex-fluids, viscoelasticity, shear-banding, yield-stress, elastoviscoplasticity

## Abstract

In this work, we implement models that are able to describe complex rheological behaviour (such as shear-banding and elastoviscoplasticity) in the *HiGTree/HiGFlow* system, which is a recently developed Computational Fluid Dynamics (CFD) software that can simulate Newtonian, Generalised-Newtonian and viscoelastic flows using finite differences in hierarchical grids. The system uses a moving least squares (MLS) meshless interpolation technique, allowing for more complex mesh configurations while still keeping the overall order of accuracy. The selected models are the Vasquez-Cook-McKinley (VCM) model for shear-banding micellar solutions and the Saramito model for viscoelastic fluids with yield stress. Development of solvers and numerical simulations of inertial flows of these models in 2D channels and planar-contraction 4:1 are carried out in the *HiGTree/HiGFlow* system. Our results are compared with those predicted by two other methodologies: the OpenFOAM-based software *RheoTool* that uses the Finite-Volume-Method and an in-house code that uses the Vorticity-Velocity-Formulation (*VVF*). We found an excellent agreement between the numerical results obtained by these three different methods. A mesh convergence analysis using uniform and refined meshes is also carried out, where we show that great convergence results in tree-based grids are obtained thanks to the finite difference method and the meshless interpolation scheme used by the *HiGFlow software*. More importantly, we show that our methodology implemented in the *HiGTreee/HiGFlow* system can successfully reproduce rheological behaviour of high interest by the rheology community, such as non-monotonic flow curves of micellar solutions and plug-flow velocity profiles of yield-stress viscoelastic fluids.

## 1. Introduction

A large majority of fluids that are of great interest in the industry (for instance, in food, pharmaceutic, plastic, oil and gas industries) exhibit viscoelastic behaviour, i.e., they show both viscous and elastic responses to forces, and thus, their characterisation is essential to estimate the ideal conditions to pump, mix and store them in industrial operations [1,2,3,4,5]. One of the key features of viscoelastic fluids is the presence of memory; stresses in such fluids depend on the flow history. In addition, they generate stresses absent in their Newtonian counterpart, resulting in interesting but complex flow phenomena.

More interestingly, complex fluids can also simultaneously display multiple rheological behaviours. An example of this kind of fluid is a *structured fluid*, i.e., those materials that contain more than one phase, such as solid particles dispersed in a liquid, suspensions, and surfactant solutions, among others, whose complex behaviour is generally dominated by the interactions between the components of the fluid. The most common example of a structured fluid is a *micellar solution* (or surfactant solutions), which consists of a dispersion of micelles in a solvent. These solutions have been studied theoretically and experimentally over the last years by the rheology community, and they are also heavily used in the pharmaceutical, cosmetic and food industries. In addition, the rheological behaviour of the micellar solutions makes them highly attractive in the industry, especially in oil-recovery processes and drilling operations, since there is a need for drilling fluids, which are specially designed fluids circulated through a wellbore as the wellbore is being drilled to facilitate the drilling operation.

When surfactant molecules (which have a hydrophilic group (water-loving) that is chemically bonded to a hydrophobic group (water-hating)) are in solution, they will self-assemble into aggregates such as spherical and wormlike micelles, bilayers, among others. Entangled solutions of wormlike micelles exhibit viscoelastic effects. However, they show a special characteristic: at very low shear rates, their shear viscosity is constant, but more importantly, they are characterised by a single stress relaxation time (unlike some polymer solutions that exhibit a spectrum of relaxation times), yielding a near-Maxwell behaviour. At higher shear rates, the entanglements may begin to break, so to model this complex fluid, it is necessary to account for the reversible assembly and disassembly of the entangled wormlike-chain solution, which is usually modelled using a kinetic equation or a mass-balance equation [6,7,8,9].

Apart from viscoelasticity, these solutions can show another fascinating rheological property. For instance, the steady, simple shear flow of a (initially entangled) wormlike micellar solution is studied. At very low shear rates ($\dot{\gamma }\ll {\dot{\gamma }}_{1}$), the fluid exhibits a linear dependence of the shear stress on the shear rate (i.e., Newtonian-like behaviour with high viscosity). An increase in $\dot{\gamma }$ will cause a drop in viscosity (shear-thinning), as viscosity strongly depends on the applied shear rate.

**Figure 1:**
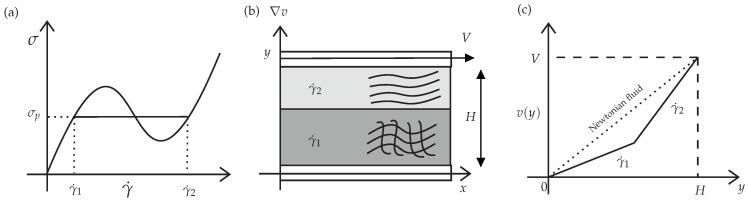
Shear-banding flow. (**a**): flow curve σ vs. $\dot{\gamma }$; (**b**): steady shear flow; (**c**): velocity profile v(y) vs. *y*.

Above a critical value of the shear stress, the initially homogeneous flow becomes unstable, which will lead the system to separate into two bands with different internal structures and different shear-rates values, ${\dot{\gamma }}_{1}$ and ${\dot{\gamma }}_{2}$, see Figure 1. These two bands are separated by an interface whose normal is in the flow-gradient direction. This phenomenon is called *shear-banded* flow or *shear-banding* transition, which describes a transition between a homogeneous and non-homogeneous state. This rheological phenomenon is analogous to the liquid-gas transition described by Van-der-Waals [6]. At very high shear rates ($\dot{\gamma }\gg {\dot{\gamma }}_{2}$), the theoretical models predict that the flow becomes homogeneous again, and the fluid will display a single but smaller viscosity [7].

The critical stress value described above is called the *stress plateau* $\sigma_{p}$: below this value, we observe entangled networks of micellar solutions; when the stress applied is $\sigma_{xy}=\sigma_{p}$, separation of bands occurs, and they coexist at this point, and above this stress value, we can idealise that the majority of entanglements will be destroyed, leading to flow-oriented linear wormlike chains. The coexistence of the low and high viscosity bands has been confirmed by different experimental techniques [10,11,12,13].

The non-monotonic curve shown in Figure 1a, which is the theoretical curve predicted by many constitutive equations, has interesting features: (1) in the banded region (${\dot{\gamma }}_{1}<\dot{\gamma }<{\dot{\gamma }}_{2}$), a negative slope for the flow curve σ vs. $\dot{\gamma }$ is seen, and homogeneous flow is unstable there, triggering the formation of bands with different shear rate values, and (2) a *multivalued region* (i.e., three different possible values of shear rate for a given value of shear stress). However, experiments have observed that real systems have well-defined stress, which is the stress plateau $\sigma_{p}$. Thus, the *real* shear flow curve is composed of two increasing stable homogeneous curves of high and low viscosities separated by a stress plateau (horizontal line) extending between two shear rate values ${\dot{\gamma }}_{1}$ and ${\dot{\gamma }}_{2}$.

The modelling of shear-banding behaviour becomes challenging, but fortunately, several different constitutive models have captured this flow curve. As Fielding [14] states, these models can be divided into two classes: (a) *phenomenological* models, which can capture the relevant physics of the phenomenon while using as few parameters as possible, and (b) models that are derived by considering the dynamics of the molecular chains, i.e., flow-induced changes on the structural level of the fluid. One of the most popular phenomenological models is the Diffusive Johnson-Segalman (DJS) model [15,16], used to describe shear-banding in dilute solutions of wormlike micelles. The stress tensor of the JS model comprises the pressure, Newtonian solvent stress and non-Newtonian stress. The last stress term is governed by a Gordon-Schowalter convected time derivative [17].

On the other hand, several models in the literature do consider these kinetic processes. A common approach taken by some of the models found in the literature is to combine dumbbell models and network theory by coupling viscoelastic governing equations and kinetic equations to break and reformate micellar solutions. One of the most popular models is the Bautista-Manero-Puig (BMP) model [7,18,19], which consists of a codeformational Maxwell constitutive equation coupled to an evolution equation for the internal structural level of the fluid [20]. This model can accurately capture complex rheological behaviour, such as shear-thinning and shear-thickening effects, thixotropy, viscoelasticity, plasticity and the shear-banding phenomenon. Another constitutive equation used to model wormlike micellar solution’s rheological behaviour was developed by Vazquez et al. [8,9], which is a *two-species reptation-reaction network* model that incorporates the reformation and continuous rate-dependent breakage of the entangled viscoelastic network. More specifically, they consider long elastic chains that can cause each break to form two short chains, which can also recombine to form the long chain. This model can describe linear and non-linear rheological behaviour before the onset of shear-banding. It also works well in predicting start-up shearing flow and cessation of steady shear flow.

Another rheological behaviour of interest in the industry is *plasticity* or *yield-stress fluid* flows, which occur in many operations and unit processes within the oil and gas industry [5], such as reservoir flows of visco-plastic heavy oils, drilling operations, wellbore cementing, among many others. In a few words, yield stress $\tau_{0}$ is defined as the minimum stress value applied to a fluid before it starts to flow. If the applied stress is smaller than the yield stress, the fluid will behave like a rigid solid (or material with extremely high viscosity). One of the simplest models that describe this behaviour is the *Bingham model*, which strongly influences the viscoplasticity literature since many more elaborated models have been derived using the ideas of Bingham. The Bingham model states that a fluid initially resists flowing until the shear stress exceeds the yield stress value. Once this condition is satisfied, the fluid will flow as a Newtonian-like fluid (constant viscosity). In the Bingham model, the solid-like and the liquid-like Newtonian contribution are combined as additive parts of the total stress. The Newtonian fluid contribution can also be replaced by a non-linear function of the shear rate; for instance, if a power-law dependence with the strain rate is assumed, then a Herschel-Bulkley model with yield stress is derived, which is one of the most representative and most used constitutive equations to describe viscoplasticity.

More complex yield-stress fluid models have been developed over the last years, which have also incorporated other rheological phenomena, such as the model proposed by Mujadmar et al. [21] (which incorporates thixotropy to a yield-stress equation), or the model of Souza Mendes et al. [22], a thirteen-parameter model commonly used in the oil and polymer industry due to its ability to model multiple rheological flows (visco-elasto-thixotropic materials with yield stress).

A simpler model for elastoviscoplastic fluids was proposed by Saramito [23], which extends both the Bingham and the Oldroyd-B viscoelastic models. One year later, Saramito [24] extended his previous model, where he derived a model that combines the Oldroyd-B model with a Herschel-Bulkley viscoplastic model with a power-law index. His general equation also allows the incorporation of viscoelastic behaviour predicted by other classic models, such as the Phan–Thien–Tanner (PTT) models.

It is of great interest for researchers and professionals in the industry to be able to carry out numerical and computational simulations of complex fluids that exhibit some of the rheological behaviour described above since it will allow us to study how these fluids behave under some specific flow scenarios and thus, reliable and efficient tools are needed. Simulation of viscoelastic fluids has been the centre of attention for many years since the mathematical complexity of their constitutive equations (non-linearity, time dependence of the stress) has been a real challenge. Efforts have been made by engineers and pure and applied mathematicians in the field of fluid mechanics and numerical analysis that have led to the development of numerical methods used to solve the governing equations of viscoelastic flows, which have also been successfully implemented in Computational Fluid Mechanics (CFD) software. This software (both commercial and open-source) allows users to simulate Newtonian and Generalised Newtonian (GNF) fluids and multi-phase and viscoelastic flows. Many of these packages for CFD are traditionally based on the Finite-Volume-Method (FVM) approach to discretise the governing equations (continuity, Navier-Stokes and constitutive equations), where the physical domain or mesh of interest needs to be divided into discrete cells/volumes. In FVM, the governing equations are integrated over a volume or cell, and a flux balance of the properties (velocity, temperature, concentration, etc.) across the boundaries of the individual volume is needed to be made. The flux of these properties is calculated at the mid-point between the discrete nodes in the domain. Hence, the flux between all neighbouring nodes in the domain can be calculated. This calculation is straightforward in a regular mesh (the same number of divisions in any direction). However, the balance of fluxes can become harder to deal with in irregular meshes. As we know, the mesh’s quality affects the solution’s accuracy and stability, and the local accuracy of the finite-volume method, such as close to a region of interest, can be increased by refining the mesh around that region. However, one of the problems of the FVM methods is that the functions that approximate the solution cannot be easily made of higher order.

Recently, F.S. Sousa et al. [25] proposed a new approach to discretise the governing equations of fluids, where they introduced a finite difference method with meshless interpolations in tree-based hierarchical grids. These Cartesian grids bring the flexibility and accuracy of local mesh refinement and allow for the development of finite difference or finite volume methods without the hassle of mapping and transforming distorted elements or dealing with general and complicated stencils, as happens in non-Cartesian grids. As discussed in [25], one of the main challenges is to adapt the discretisation stencil near the interfaces between grid elements of different sizes, which is usually solved by local high-order geometrical interpolations. These interpolations, however, depend on the distribution of cells in the vicinity of the point of interest. Hence they are site-specific and can become challenging to calculate in three-dimensional simulations, especially when dealing with staggering arrangements (where some scalar variables are stored in cell centres, whereas the velocities are located at the cell facets). Most methods tend to avoid this by limiting the mesh configuration (usually to graded quadtree/octree grids), thus, reducing the number of cases to be treated locally. The interpolations used in their numerical method are based on Moving Least Squares (MLS) approximations performed to compute the final finite difference stencil weights, allowing for complex mesh configurations while still keeping the overall order of accuracy of the resulting method. This numerical method has already been tested in different flow configurations, and the results were compared with already published methods [25,26,27], showing excellent accuracy and flexibility of this new methodology.

This new numerical method was used to develop the *HiGTree/HiGFlow* system (HiG stands for hierarchical grids) by the same research group, a CFD software that allows simulating Newtonian, GNF, multi-phase, electroosmotic and viscoelastic flows. Some of the classic and most popular viscoelastic models (such as Oldroyd-B, Giesekus, linear-PTT, generalised-PTT and KBKZ-integral models) are already available in the *HiGFlow* software as well as viscoelastic models that incorporate non-constant viscosity and time-dependent phenomena (such as thixotropy). Thus, our goal in this work is to implement new models into the *HiGTree/HiGFlow* system that can describe more complex rheological behaviour, such as shear-banding and elastoviscoplasticity (yield stress fluids). The selected models to be implemented are the Vasquez-Cook-Mckinley [8,9] and the Saramito [24] models. To verify the simulations carried out with *HiGFlow*, our results will be compared with solutions predicted by two different methodologies: (1) the *OpenFOAM/RheoTool* system [28], an open-source CFD software that uses the FVM approach to discretise the governing equations and where the VCM and Saramito models are already implemented; and (2) the Vorticity-Velocity-Formulation (*VVF*). This will allow us to demonstrate that our solvers implemented in *HiGFlow* can successfully predict rheological behaviour of interest, such as non-monotonic curves of micellar solutions, non-zero normal stresses, and plug-flow velocity profiles of viscoelastic fluids with yield-stress.

The structure of this work is as follows: in Section 2, we show the general governing equations and the dimensionless groups adopted to simulate viscoelastic fluids exhibiting shear-banding and plasticity. In Section 3, we describe the numerical methods used to simulate flows using the *HiGTree/HiGFlow* system, the *RheoTool* and the Vorticity-Velocity Method. Lastly, our numerical results are illustrated in Section 4, where we describe the interpolation and discretisation schemes to be used in our simulations, as well as the details of the geometries/meshes and model parameter values, and the comparison between our *HiGFlow*, *RheoTool* and our in-house *VVF* results. A discussion of the physical interpretation of the results obtained is also presented in each subsection.

## 2. Governing Equations

This section shows the general governing equations in the tensorial form adopted in the present paper and implemented in the adopted codes. In a later Section 3, we show the discretised form of these equations.

For transient, isothermal and incompressible flows, the mass conservation and momentum equations (in the absence of external forces such as gravity) are:

(1) ∇·u=0,

(2) $$\rho \left(\frac{\partial u}{\partial t}+u\cdot \nabla u\right)=-\nabla P+2\;\eta_{s}\;\nabla \cdot D+\nabla \cdot \tau ,$$

(3) $$\tau =\tau \left(\eta ,D\right),$$

where u is the velocity field, *t* is the time, ρ is the fluid density, *P* is the pressure, $\eta_{s}$ is the solvent viscosity, and τ is a viscoelastic stress tensor (see Equation (3)) that can depend on the viscosity and on the deformation tensor D, which is defined as:

(4) $$D=\frac{1}{2}\left[\nabla \;u+\nabla \;u^{⊤}\right].$$

### 2.1. The Both-Sides-Diffusion (BSD) Technique

For our numerical methods, we will implement the both-sides-diffusion (BSD), which is a technique that consists in adding a diffusive term on both sides of the momentum equation. Once a steady state is reached, both terms cancel each other exactly. Such a method increases the ellipticity of the momentum equation and has a stabilising effect. Incorporating the terms arising from the both-sides-diffusion in the momentum Equation (2) and rearranging the equations, we have:

(5) $$\rho \left(\frac{\partial u}{\partial t}+u\cdot \nabla u\right)-2\;[\eta_{s}+\eta_{0}]\;\nabla \cdot D=-\nabla P-2\;\eta_{0}\;\nabla \cdot D+\nabla \cdot \tau ,$$

As it can be seen, we have introduced a diffusive term of the form $2\eta_{0}\nabla \cdot D$ on both sides of the equation. Here, $\eta_{0}$ is the characteristic viscosity of the polymer, which can vary from fluid to fluid, and it will depend on the selected model by the user. In most cases, $\eta_{0}$ is the viscosity at low-shear rates, which can be easily measured experimentally.

### 2.2. The Vasquez-Cook-McKinley (VCM) Model

In 2007, the Vasquez-Cook-McKinley (VCM) [8] model was published to describe the rheological behaviour of micellar solutions that exhibit shear-banding. Their model is based on the “living polymer theory” proposed by Cates. In order to derive the VCM model, they considered two active Hookean species: long chains can break to form short chains, which can recombine to form a long chain. The chains undergo rupture at a rate dependent on the local elongation and deformation rate.

**Figure 2:**
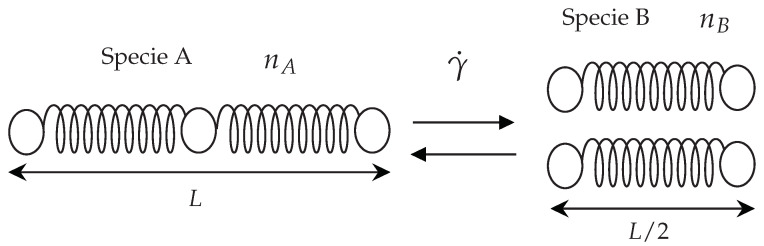
Visual representation of the process of breakage and reformation of the micellar chains modelled by the VCM model: the specie A (long chains) can break to form short chains (specie B), which can themselves recombine to form a long chain (specie A). The chains undergo rupture at a rate dependent on the local elongation and deformation rate $\dot{\gamma }$.

The model (see Figure 2) represents the micellar solutions as a combination of large (subscript A) and small chain (subscript B) species that can convert into each other. A transport equation is solved for each species using the following equations

(6) $$\frac{\partial n_{A}}{\partial t}+u\cdot \nabla \;n_{A}=2D_{A}\;\nabla^{2}\;n_{A}+\frac{1}{2\;\lambda_{A}}c_{B}\;n_{B}^{2}-\frac{c_{A}\;n_{A}}{\lambda_{A}},$$

(7) $$\frac{\partial n_{B}}{\partial t}+u\cdot \nabla \;n_{B}=2D_{B}\;\nabla^{2}\;n_{B}-\frac{c_{B}\;n_{B}^{2}}{\lambda_{A}}+2\;\frac{c_{A}\;n_{A}}{\lambda_{A}},$$

where $n_{i}$ is the dimensionless number density of the specie, $\lambda_{i}$ is the relaxation time, $D_{i}$ is the diffusivity coefficient, $c_{A}$ and $c_{B}$ are, respectively, the dimensionless breakage and reformation rates, which can be calculated as follows:

(8) $$c_{A}=c_{A_{Eq}}+\frac{\chi }{3}\left(\dot{\gamma }:\frac{A}{n_{A}}\right),$$

(9) $$c_{B}=c_{B_{Eq}}.$$

In Equation (8), the longer elastic segments (specie A) will experience convection by the flow and recoil following a breakage event before being reincorporated into the network, where $c_{A_{Eq}}$ and $c_{B_{Eq}}$ are equilibrium concentrations, χ is a parameter associated to the structural destruction, and $\dot{\gamma }$ is a symmetric tensor:

(10) $$\dot{\gamma }=\left[\nabla \;u+\nabla \;u^{⊤}\right].$$

In Equations (6) and (7), the double contraction term presented in the original paper [8] is omitted here in our equations to simplify the definition of no-flux boundary conditions for the density number at the walls, which reduce to a zero-gradient condition. More importantly, we will be focusing on simulating inertial flows (Re≠0); therefore, the contribution of these omitted terms is negligible.

A viscoelastic constitutive equation is also solved for each specie:

(11) $$\overset{\nabla }{A}=\frac{1}{\lambda_{A}}\left(n_{A}\;I-A\right)+\frac{1}{\lambda_{A}}\left(c_{B}\;n_{B}\;B-c_{A}\;A\right)+D_{A}\;\nabla^{2}\;A,$$

(12) $$\overset{\nabla }{B}=\frac{1}{\lambda_{B}}\left(\frac{n_{B}}{2}\;I-B\right)+\frac{2}{\lambda_{A}}\left(c_{A}\;A-c_{B}\;n_{B}\;B\right)+D_{B}\;\nabla^{2}\;B.$$

The identity tensor is represented as I, A and B represent the conformation tensor of each species, and the hat symbol ∇ represents the upper-convected-derivative of a tensor, which is expressed below in terms of the tensor A:

(13) $$\overset{\nabla }{A}=\frac{DA}{Dt}-A\cdot \left(\nabla \;u\right)-{\left(\nabla \;u\right)}^{⊤}\cdot A.$$

Finally, the contribution of each species to the polymeric extra-stress tensor is given by:

(14) $$\tau =G_{0}\left[\left(A+2\;B\right)-\left(n_{A}+n_{B}\right)\;I\right],$$

where $G_{0}$ is the elastic modulus.

### 2.3. Dimensionless Form of the Governing Equations for Viscoelastic Micellar Solution Flows Using the VCM Model

In order to obtain the dimensionless governing equations that we will be using to simulate VCM fluids, we will use the same scales reported in [29]; we scale times with the effective (or overall) relaxation time of the network $\lambda_{eff}$, which is defined as: $\lambda_{eff}=\lambda_{A}/\left(1+C_{A_{eq}}^{\prime }\lambda_{A}\right)$; for the velocities, we we use a characteristic velocity $L/\lambda_{eff}$; we scale stresses with a convective characteristic stress defined as $\rho \;{\left(L/\lambda_{eff}\right)}^{2}$, and lastly, we scale viscosities with $\eta_{0}=\lambda_{eff}G_{0}$. Thus, the mass and momentum conservation equations become:

(15) ∇·u=0,

(16) $$\frac{\partial u}{\partial t}+u\cdot \nabla u=-\nabla P+2\;E\;\left(1+\frac{\beta }{1-\beta }\right)\nabla \cdot D+\nabla \cdot S.$$

In these equations, we define the polymeric tensor S (which is a direct result of implementing the both-sides-diffusion technique) and the dimensionless stress tensor:

(17) $$S=T-2\;E\;D\;T=E\left[\left(A+2B\right)-\left(n_{A}+n_{B}\right)I\right].$$

We can also notice that we have introduced two dimensionless parameters, *E* and β, the elasticity number and the solvent viscosity ratio, respectively, which are:

(18) $$E=\frac{\eta_{0}\;\lambda_{eff}}{\rho \;L^{2}}=\frac{De_{eff}}{Re_{V}}\;\beta =\frac{\eta_{s}}{\eta_{s}+\eta_{0}},$$

As seen in Equation (18), the elasticity number *E* can be expressed as the ratio between the efficient Deborah number of the network $De_{eff}$ and the Reynolds number ($Re_{V}$), which we conveniently defined as shown below:

(19) $$De_{eff}=\lambda_{eff}\;\left(\frac{U_{0}}{L}\right)=\frac{\eta_{0}}{G_{0}}\left(\frac{U_{0}}{L}\right)\;Re_{V}=\frac{\rho \;U_{0}\;L}{\eta_{0}},$$

where $U_{0}$ is a characteristic velocity associated with the flow, which is different to the velocity we previously defined in terms of the efficient relaxation time ($L/\lambda_{eff}$). Notice that $U_{0}$ cancels out when we calculate *E*. We found it very convenient to dimensionalise our governing equations using $L/\lambda_{eff}$ instead of $U_{0}$ because it allows us to stabilise the numerical simulations of this flow since we require high-velocity values in order to observe the shear-banding behaviour of micellar solutions.

On the other hand, the dimensionless viscoelastic equations for the conformation tensors A and B and the conservation equations for the density numbers of species *A* ($n_{A}$) and species *B* ($n_{B}$) are:

(20) $$\overset{\nabla }{A}=\frac{1}{De_{A}}\left(n_{A}\;I-A\right)+\frac{1}{De_{A}}\left(c_{B}\;n_{B}\;B-c_{A}\;A\right)+\frac{1}{Pe_{A}}\;\nabla^{2}\;A,$$

(21) $$\overset{\nabla }{B}=\frac{1}{De_{B}}\left(\frac{n_{B}}{2}\;I-B\right)+\frac{2}{De_{A}}\left(c_{A}\;A-c_{B}\;n_{B}\;B\right)+\frac{1}{Pe_{B}}\;\nabla^{2}\;B,$$

(22) $$\frac{\partial n_{A}}{\partial t}+u\cdot \nabla \;n_{A}=\frac{2}{Pe_{A}}\;\nabla^{2}\;n_{A}+\frac{1}{2\;De_{A}}c_{B}\;n_{B}^{2}-\frac{c_{A}\;n_{A}}{De_{A}},$$

(23) $$\frac{\partial n_{B}}{\partial t}+u\cdot \nabla \;n_{B}=\frac{2}{Pe_{B}}\;\nabla^{2}\;n_{B}-\frac{c_{B}\;n_{B}^{2}}{De_{A}}+2\;\frac{c_{A}\;n_{A}}{De_{A}},$$

with breakage and reformation rates for $c_{A}$ and $c_{B}$ that are calculated using the expressions already reported in Equations (8) and (9), considering that our parameter associated to the breakage rate χ is $\chi =(\lambda_{A}/\lambda_{eff})\xi$, see [29].

Four new dimensionless groups were derived; the first two are what we call the Deborah numbers of species *A* ($De_{A}$) and species *B* ($De_{B}$), which are:

(24) $$De_{A}=\left(\frac{\lambda_{A}}{\lambda_{eff}}\right)\;De_{B}=\left(\frac{\lambda_{B}}{\lambda_{eff}}\right).$$

We also have defined two Péclet numbers, one for each specie, which compare the transport of species by convection with the transport by diffusion:

(25) $$Pe_{A}=\left(\frac{D_{A}\lambda_{eff}}{L^{2}}\right)\;Pe_{B}=\left(\frac{D_{B}\lambda_{eff}}{L^{2}}\right).$$

Generally, we will assume that these numbers tend to be large and that $Pe_{A}=Pe_{B}$. Thus, our final set of Equations (15)–(25) are implemented in the *HiGTree/HiGFlow* system, which will be then solved to simulate flows of micellar solutions that exhibit shear-banding.

### 2.4. The Saramito Model

Elastoviscoplastic fluids exhibit a solid-like behaviour below critical stress, commonly known as *yield stress* $\tau_{0}$, and they flow as viscoelastic fluids when the yield stress is exceeded. In 2009, Saramito [23,24] proposed a model that combines the viscoplastic and yield stress effects from the Herschel–Bulkley model with the viscoelastic behaviour predicted by classical models such as the Oldroyd-B and Phan–Thien–Tanner (PTT). Accordingly, his constitutive equation adopts the following general form:

(26) $$f\left(\tau \right)\;\eta_{P}\;\max{\left(0,\;\frac{\bar{\sigma }-\tau_{0}}{k\;{\bar{\sigma }}^{\;n}}\right)}^{\frac{1}{n}}\tau +\lambda_{p}\overset{□}{\tau }=2\;\eta_{P}\;D,$$

where $\overset{□}{\tau }$ is the Gordon-Schowalter derivative defined as:

(27) $$\overset{□}{\tau }\;=\;\overset{\nabla }{\tau }+\;\xi \;\left(\tau \cdot D+D\cdot \tau \right).$$

The slip-parameter ξ takes into account the non-affine motion between the polymer molecules and the continuum, and if ξ=0, the Gordon-Schowalter derivative reduces to the upper-convected-derivative, which is defined below:

(28) $$\overset{\nabla }{\tau }=\frac{D\tau }{Dt}-\tau \cdot \left(\nabla \;u\right)-{\left(\nabla \;u\right)}^{⊤}\cdot \tau .$$

In Equation (26), $\eta_{P}$ is the polymer viscosity, *n* is the power index, k>0 is the consistency parameter, f(τ) is a function dependent on the stress that can be imposed on the constitutive equation to reproduce the viscoelastic behaviour observed in the Oldroyd-B model or the linear (LPTT) or exponential (EPTT) models; $\bar{\sigma }$ is a parameter that can be defined as the second invariant of the deviatoric stress tensor $\tau_{D}$:

(29) $$\bar{\sigma }=II_{\tau_{D}}=\sqrt{\frac{\tau_{D}:\tau_{D}}{2}}$$

(30) $$\tau_{D}=\tau -\frac{\mathrm{tr}(\tau )}{N}\;I,$$

where *N* is the number of dimensions in the flow problem (N=2 for a 2D flow case and N=3 for a 3D problem).

Saramito’s general constitutive Equation (26) can be reduced to a previous model proposed by the same author [23] if the parameters *n* and *k* take the following values, n=1, $k=\eta_{P}$, which will give a model that is able to merge the classic Bingham model with the Oldroyd-B or the PTT models, depending on the form that the function f(τ) takes:

(31) $$f\left(\tau \right)=\left\{\begin{array}{cc} 1 & \;\;\;\;,\;\mathrm{Oldroyd}-B\\ 1+\frac{\epsilon \;\lambda_{p}}{\eta_{P}}\mathrm{tr}\left(\tau \right) & ,\;\mathrm{linear}\mathrm{PTT}\\ \exp\frac{\epsilon \;\lambda_{p}}{\eta_{P}}\mathrm{tr}\left(\tau \right) & \;\;\;\;\;\;\;\;\;\;\;\;,\;\mathrm{exponential}\mathrm{PTT}, \end{array}\right.$$

where ϵ is the extensibility parameter.

### 2.5. Dimensionless Form of the Governing Equations for Elastoviscoplastic FLOWS Using the Saramito Model

For our equations, we scale lengths with *L*, we use the average shear rate $U_{0}L^{-1}$ to scale times (where $U_{0}$ is a characteristic velocity), and finally, we scale stresses with a characteristic convective flux ($\rho U_{0}^{2}$). Thus, the momentum and mass conservation equations can be written in dimensionless form as follows:

(32) ∇·u=0

(33) $$\frac{\partial u}{\partial t}+u\cdot \nabla u=-\nabla P+\frac{2}{Re}\nabla \cdot D+\nabla \cdot S,$$

where Re is the dimensionless Reynolds number defined as

(34) $$Re=\frac{\rho \;U_{0}\;L}{\eta_{s}+\eta_{P}}.$$

In Equation (33), we have also introduced a new tensor named the polymeric tensor, which is:

(35) $$S=T-\frac{2(1-\beta )}{Re}D,$$

where β is the dimensionless solvent viscosity ratio, $\beta =\eta_{s}/\left(\eta_{s}+\eta_{P}\right)$; ∇u and $\nabla \;u^{⊤}$ are the velocity gradient and its transpose, respectively, and T is the elastic stress tensor (the dimensionless form of the tensor τ), which can be written in terms of the conformation tensor $A_{S}$ for the Saramito model:

(36) $$T=\frac{1-\beta }{Re\;De}\left(A_{S}-I\right).$$

Here, I is the identity tensor, and De is the Deborah number, which is defined as:

(37) $$De=\lambda_{p}\;\left(\frac{U_{0}}{L}\right),$$

where $\lambda_{p}$ is the characteristic stress relaxation time of the polymer, $\lambda_{p}=\left(\eta_{P}/G_{0}\right)$ ($G_{0}$ is a constant shear-modulus). The dimensionless form of Equation (26) in terms of the dimensionless stress tensor T can be written in general form as:

(38) $$\overset{\nabla }{T}=\frac{1}{De}\left[\frac{2(1-\beta )}{Re}D-f\left(T\right)\;T-\xi \;De\;\left(T\cdot D+D\cdot T\right)\right],$$

where $f\left(T\right)$ is a stress-dependent function that allows us to implement the four variants of elastoviscoplastic models that can be described by the Saramito general equation [23,24].

In order to overcome the numerical instabilities seen at high Deborah number values, Equation (38) can be reformulated and be written as a function of the conformation tensor $A_{S}$ by using the decomposition of the velocity gradient proposed by Fattal et al. [30,31], $\nabla \;u^{⊤}=\Omega +B_{S}+N\;A_{S}^{-1}$, where Ω and N are anti-symmetric tensors, and $B_{S}$ is symmetric and commutes with $A_{S}$. As a result, Equation (38) becomes:

(39) $$\overset{\nabla }{A_{S}}=\frac{1}{De}\;\left[M\left(\sigma_{d}\right)\;\left(I-A_{S}\right)-f\left(\xi \right)\right].$$

where $M\left(\sigma_{d}\right)$ can adopt each of the following forms:

(40) $$M\left(\sigma_{d}\right)=\left\{\begin{array}{cc} \max\left(0,\;\frac{\sigma_{d}-Bi}{\sigma_{d}}\right) & \;\;\;\;\;\;\;\;\;,\;\mathrm{Oldroyd}-B-\mathrm{Bingham}\\ \max{\left(0,\;\frac{\sigma_{d}-Bi}{K\;\sigma_{d}^{n}}\right)}^{\frac{1}{n}} & ,\;\mathrm{Oldroyd}-B-\mathrm{HB}\\ \max\left(0,\;\frac{\sigma_{d}-Bi}{\sigma_{d}}\right)\;\left[1+\frac{\epsilon \;De\;Re\;\mathrm{tr}(S)}{1-\beta }\right] & ,\;\mathrm{LPTT}-\mathrm{Bingham}\\ \max\left(0,\;\frac{\sigma_{d}-Bi}{\sigma_{d}}\right)\;\exp\left[\frac{\epsilon \;De\;Re\;\mathrm{tr}(S)}{1-\beta }\right] & ,\;\mathrm{EPTT}-\mathrm{Bingham}, \end{array}\right.$$

with:

(41) $$f\left(\xi \right)=\xi \;De\;\left(B_{S}-B_{S}\cdot A_{S}\right)],$$

which is a term that takes into account non-affine motion. In Equation (40), we show the four viscoelastic variants that can be simulated using the Saramito’s general equation: the Oldroyd-B-Bingham, the Oldroyd-B-Herschel-Bulkley, the liner-PTT-Bingham and the exponential-PTT-Bingham. The dimensionless parameters that appear in this equation are: $\sigma_{d}$, which is defined as $\sigma_{d}=\sqrt{(T_{D}:T_{D})/2}$, where $T_{D}$ is the dimensionless form of the deviatoric stress tensor, $T_{D}=T-\left(\mathrm{tr}\left(T\right)/N\right)I$, *K* is the dimensionless consistency parameter, $K={\left[Re/\left(1-\beta \right)\right]}^{1-n}$ and Bi is our Bingham number:

(42) $$Bi=\frac{\tau_{0}}{\rho \;U_{0}\;U_{0}}.$$

If the stress $\sigma_{d}$ is below Bi, the fluid will behave as a rigid-solid. On the other hand, if $\sigma_{d}>Bi$, the material will flow as a viscoelastic fluid.

In addition, notice that if the power-law index n=1 that appears as a model parameter in the Oldroyd-B-Herschel-Bulkley equation, we recover the original Oldroyd-B-Bingham model; if n<1, the fluid will exhibit viscoelastic shear-thinning behaviour (if $\sigma_{d}>Bi$) and we will observe shear-thickening behaviour for n>1 (if $\sigma_{d}>Bi$).

Recently, Afonso et al. [32] proposed a generic *Kernel-conformation* tensor transformation for a large class of differential constitutive models, in which the evolution equation of the kernel tensor $k\left(A_{S}\right)=Ok\left(\Lambda \right)O^{T}$ (where k represents any continuous, invertible and differentiable matrix transformation function and O is a matrix containing in its columns the eigenvectors of $A_{S}$), can be expressed in its tensorial formulations as:

(43) $$\frac{Dk(A_{S})}{Dt}=\Omega \;k\left(A_{S}\right)-k\left(A_{S}\right)\;\Omega +2\;B+\frac{1}{De}\;M,$$

In Equation (43), B and M are symmetric tensors constructed by the orthogonalization of the diagonal tensors $D_{B}$ and $D_{M}$, respectively (see their definition and a full-detailed explanation of this approach in [26]).

The set of Equations (32)–(43) were implemented in the *HiGTree/HiGFlow* system, which will allow us to simulate different elastoviscoplastic flow scenarios.

## 3. Numerical Method

Three different codes were used in the present study: *HiGTree/HiGFlow, RheoTool*, and Vorticity-Velocity code. Each code has its features; therefore, each one is described below.

### 3.1. HiGTree/HiGFlow

In this section, we will give a brief introduction to the *HiGFlow* system. *HiGFlow* (HiG stands for hierarchical grids) is a Computational Fluid Dynamics (CFD) software written in C language that was developed at the Institute of Mathematics and Computer Sciences (ICMC) from the University of São Paulo (USP). The system can simulate single and multi-phase flows of Newtonian and Non-Newtonian fluids using a new finite difference method [25] that was recently published to solve partial differential equations derived from Newtonian incompressible flows. This system is being developed modularly, allowing new techniques and methods to be easily tested and implemented.

Some of the main features of *HiGFlow* are: the user can choose the dimension (2D or 3D) and the modules to be used in the program (such as single- or multi-phase, Newtonian, generalised Newtonian or viscoelastic flows) at compile-time. In the same way, the user specifies the numerical techniques to be used in the input files: for instance, projection method (incremental or non-incremental), temporal discretisation methods (explicit or semi-implicit Euler, third order Runge-Kutta, Crank-Nicolson or Backward Differentiation Formula), the numerical scheme for the convective and diffusive terms (1st and second order central differencing scheme, first order Upwind, second order Quick, CUBISTA, etc.), the constitutive equation for viscoelastic flows (such as Oldroyd-B, Giesekus, EPTT and GPTT models), in addition to the various parameters for simulation (i.e., Reynolds and Deborah numbers, etc.).

On the other hand, the HiGTree system is responsible for creating the data structure (hierarchical grid), domains, and linear and non-linear system solvers, as well as carrying out the interpolations schemes (see Section 3.1.1). Parallelisation strategies are also implemented through the PETSc library (Portable, Extensible Toolkit for Scientific Computation), which contains a set of functions implementing the best-known methods for representing matrices, vectors and data in parallel, solution of linear systems with pre-conditioning, solution of linear and non-linear systems, ordinary differential equations, etc.

#### 3.1.1. Hierarchical Grids and Meshless Interpolations

**Figure 3:**
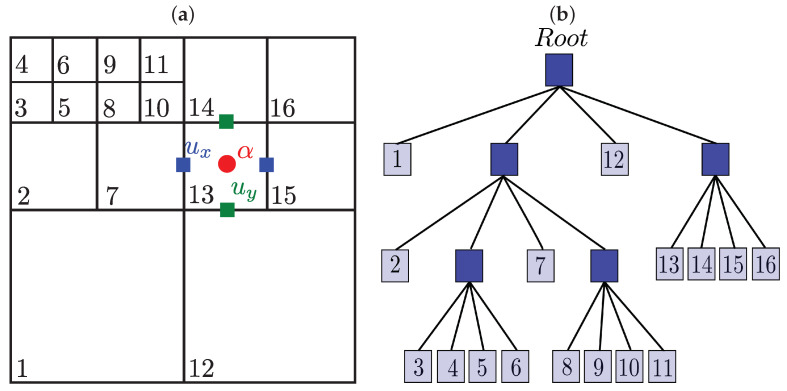
HiGTree data structure: (**a**) Computational cell representation, (**b**) Tree-based data structures.

Here, we will briefly describe the basic concepts of the finite difference techniques in hierarchical cartesian meshes already implemented in *HiGFlow*. In Figure 3, we illustrate an example of a hierarchical grid and the tree data structure representing it. This kind of structure has been used in previous works to solve the Navier-Stokes equations, where a staggered grid arrangement of the unknowns allows a stable discretisation for uniform grids. In this arrangement, components of the velocity *u* are evaluated at the cell facets (green and blue squares). In contrast, scalar and tensorial quantities represented by α (such as pressure, stress tensor, densities, etc.) are evaluated at the cell centres (red circle).

In this data structure, each cell can be geometrically partitioned in any matrix arrangement of cells. The problem, however, is that such general grids impose difficulties in the numerical approximation in finite differences approximation. This is because they usually require the computation of spacial interpolations in the unknown points of the finite difference stencil that heavily relies on the geometrical characteristics of the grid. In order to avoid this geometrical dependence, F.S. Sousa et al. [25] introduced a method based on interpolations in a neighbouring point cloud, requiring no geometry or topological information that is performed by an efficient Moving-Least-Square (MLS) interpolation scheme.

**Figure 4:**
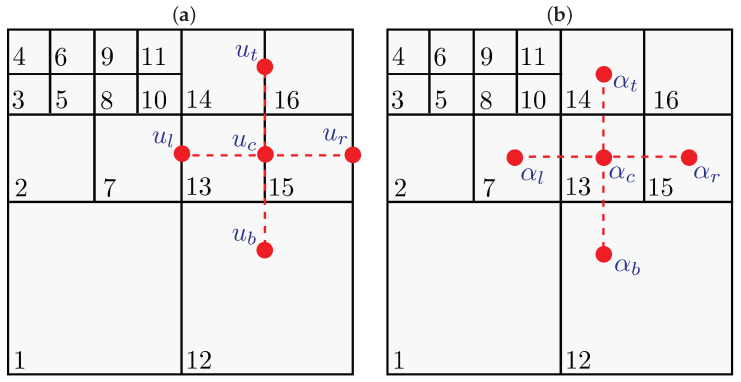
Finite difference second order stencil discretisation. The figures show the discretisation of the (**a**) velocity (evaluated at the facet centres) and (**b**) of the scalar and tensorial quantities α (evaluated at the cell centres). Notice that the variables $u_{b}$ and $\alpha_{b}$ do not coincide with the mesh grid points.

To illustrate an example of this, let us consider Figure 4, and suppose that we are interested in approximating the second derivative of a variable *u* in *y*-direction on the point *c*. Using second-order finite differences, we have:

(44) $$\frac{\partial^{2}u}{\partial y^{2}}\approx \frac{1}{\Delta y}\left(u_{t}-2u_{c}+u_{b}\right).$$

It can be easily noticed from Equation (44) and Figure 4 that $u_{b}$ does not coincide with some of the mesh grid points (recalling that the component of the velocities is evaluated at the facets centres). Thus, $u_{b}$ has to be approximated by some interpolation of the grid unknowns in the vicinity of $u_{c}$. This interpolation can be carried out as follows:

(45) $$u_{b}=\sum_{k\in V_{b}}w_{k}^{b}u_{k},$$

where $V_{b}=i_{k},k=1,\ldots ,N_{l}$ is the set of indexes for the unknowns that are in the vicinity of $u_{c}$ for each approximation. The number of neighbours $N_{l}$ is defined according to the imposed precision on the numerical method. On the other hand, the weights $w_{k}$ are computed by a Moving Least Squares (MLS) procedure. The full details of the interpolations can be found in [25].

We are interested in simulating incompressible complex fluid flows using general hierarchical tree-based meshes through the general meshless interpolation scheme described in Section 3.1.1.

As previously mentioned, the pressure values are evaluated in the cells’ centre, while the velocity components are evaluated at the cell facets. The other variables (polymeric stress and kernel tensors) will be located at the cell centres. The governing equations will then be discretised with finite difference methods in a Cartesian mesh that could have different cell sizes. As discussed before, although some of the variables required to solve the equations might be located at unknown points of the stencil, the *HiGTree/HiGFlow* can calculate the properties at those points through a function called “*compute-value-at-point*”, which will return an approximate value of the property using the Moving-Least-Square (MLS) interpolation scheme. Full details of this approach can be found in [25]. Here, we will summarise the discretisation schemes by the *HiGTree/HiGFlow* software.

The *HiGTree/HiGFlow* system uses the projection method for the momentum conservation equations. Upon discretising Equation (32) in time using a first-order implicit discretisation, the idea of this projection method is to use the newest previous pressure field, which leads to an explicitly-computed velocity field $v^{*}$ that is not divergence-free ($\nabla \cdot v^{*}\neq 0$). This velocity field $v^{*}$ can be calculated through the solution of:

(46) $$\frac{u^{*}-u^{n}}{\Delta t}=-\nabla P^{n}-u^{n}\cdot \nabla u^{n}=+a\;\nabla \cdot D^{n}+\nabla \cdot S^{n},$$

with $u^{*}$ satisfying the same boundary conditions as *u* and *a* is a constant, which depends on the model we are using; a=2/Re for the Saramito model (see Equation (33)) and a=2E(1+(β/(1−β)) for the VCM model (see Equation (16)). The corrected velocity field can be computed from a Helmholtz-Hodge decomposition to $u^{*}$:

(47) $$u^{*}=u^{n+1}+\nabla \phi ,$$

where $\phi =-\Delta t(P^{n+1}-P^{n})$, which is obtained by solving the following Poisson equation:

(48) $$\nabla^{2}\phi =\nabla \cdot u^{*}.$$

For the Poisson Equation (48) and all the terms that involve the operator ∇·, we use a second-order central differencing scheme to discretise the laplacians terms. For the viscoelastic constitutive equations of the tensors (A and B) of the VCM model (see Equations (20)–(23)), we use an explicit Euler method to discretise them:

(49) $$A^{n+1}=A^{n}+\Delta t\left[A^{n}\cdot {\left(\nabla \;u\right)}^{n}+{\left(\nabla \;u^{n}\right)}^{⊤}\cdot A^{n}-u^{n}\cdot \nabla \;A^{n}+F\left(B^{n},n_{A}^{n},n_{B}^{n}\right)\right],$$

(50) $$B^{n+1}=B^{n}+\Delta t\left[B^{n}\cdot {\left(\nabla \;u\right)}^{n}+{\left(\nabla \;u^{n}\right)}^{⊤}\cdot B^{n}-u^{n}\cdot \nabla \;B^{n}+F\left(A^{n},n_{A}^{n},n_{B}^{n}\right)\right],$$

where $F(B^{n},n_{A}^{n},n_{B}^{n})$ and $F(A^{n},n_{A}^{n},n_{B}^{n})$ are the right-hand sides of Equations (20) and (21), respectively. Similarly, we can use an explicit Euler method to discretise the density number equations for $n_{A}$ and $n_{B}$:

(51) $$n_{A}^{n+1}=n_{A}^{n}+\Delta t\left[-u^{n}\cdot \nabla \;n_{A}^{n}+F\left(n_{B},c_{A},c_{B}\right)\right],$$

(52) $$n_{B}^{n+1}=n_{B}^{n}+\Delta t\left[-u^{n}\cdot \nabla \;n_{B}^{n}+F\left(n_{A},c_{A},c_{B}\right)\right],$$

with $F(n_{B},c_{A},c_{B})$ and $F(n_{A},c_{A},c_{B})$ being the right-hand side of the conservation equations for the densities (see Equations (22) and (23)).

For the Saramito general model and the variants that can be derived from it (Oldroyd-B-Bingham, Oldroyd-B-Herschel-Bulkley, LPTT-Bingham and EPTT-Bingham), we have to discretise the governing equation in terms of the Kernel tensor k (see Equation (43)). Applying an Euler explicit method, we have:

(53) $$k^{n+1}=k^{n}+\Delta t\left[-\left(u^{n}\cdot \nabla \right)k^{n}+\Omega^{n}\;k^{n}-k^{n}\;\Omega^{n}+2\;B^{n}+\frac{1}{De}\;M^{n}\right].$$

The kernel $k^{n}$ is then used to obtain the elastic stress tensor $T^{n}$ and the polymeric tensor $S^{n}$ that is included in the momentum conservation Equations (46).

Lastly, all the convective terms from the governing equations of the VCM and Saramito fluids are discretised using a CUBISTA (Convergent and Universally Bounded Interpolation Scheme for the Treatment of Advection) scheme [33].

### 3.2. Overview of the Numerical Method

In order to summarise the numerical method, we now describe the computational steps of our solver.

(1) Initialise the fields for pressure *p*, velocity u, polymeric S and elastic stress T tensors, the kernel tensor k for the Saramito model and the conformations tensors A, B and the density number fields $n_{A}$ and $n_{B}$ for the VCM model at time t=0 and set boundary conditions.
(2) Enter the time loop (t=Δt).
  (a) Solve the Navier-Stokes equations for the velocity field $u^{*}$ using (46).
  (b) Solve the Poisson Equation (48) to obtain ϕ.
  (c) Calculate the corrected velocity field $u^{n+1}$ using (47) and $P^{n+1}$ from $\phi =-\Delta t(P^{n+1}-P^{n})$.
  (d) If solving a flow problem using the VCM model:
    (i) Compute the conformation tensors $A^{n+1}$ and $B^{n+1}$ using the discretised Equations (49) and (50).
    (ii) Compute the density numbers of the two species $n_{A}^{n+1}$ and $n_{B}^{n+1}$ using Equations (51) and (52).
    (iii) Calculate the new polymeric $S^{n+1}$ and elastic stress tensors $T^{n+1}$ using Equation (17).
  (e) If solving a flow problem using the Saramito model or the simplified forms derived from such model:
    (i) Compute the conformation tensor $A_{S}$ (Equation (39)) and their respective eigenvalues and eigenvectors and apply the kernel transformation approach to obtain the kernel tensor k.
    (ii) Solve the evolution of the kernel tensor Equation (53) to obtain $k^{n+1}$.
    (iii) Compute the inverse of the kernel tensor and calculate the new conformation $A_{S}^{n+1}$, polymeric $S^{n+1}$ and elastic stress tensor $T^{n+1}$ with Equation (35).
  (f) Update all the fields to be used in the next time loop:
    ${\left\{p,u,S,A,B,T,n_{A},n_{B}\right\}}_{n}={\left\{p,u,S,A,B,n_{A},n_{B}\right\}}_{n+1}$ for the VCM model, and ${\left\{p,u,S,T,k\right\}}_{n}={\left\{p,u,S,T,k\right\}}_{n+1}$ for the Saramito model.
(3) Increment the time t=t+Δt and return to step 2 until the final time is reached.

### 3.3. * RheoTool*

In order to compare and validate the results of our numerical simulations obtained using the *HiGTree/HiGFlow* system, we will also carry out flow simulations in the *RheoTool* system [28]. *RheoTool* is an open-source toolbox based on OpenFOAM to simulate Generalised Newtonian Fluids (GNF) and viscoelastic fluids under pressure-driven and/or electrically-driven flows. In the present work, we use the OpenFOAM version 7.0 together with *RheoTool* version 5.0.

Some of the many features of *RheoTool* are: (1) a logarithm transformation of the conformation tensor is implemented, allowing to reach higher Deborah numbers without loss of positive definiteness of the conformation tensor, (2) several techniques are available for stabilisation purposes (for instance, the both-sides-diffusion technique, the pressure-velocity and stress-velocity coupling algorithms), (3) high-resolution schemes for the discretisation of convective terms, (4) a constitutive equations library that contains a large amount of viscoelastic and GNF models (including the original BMP model), (5) it includes interfaces to the sparse matrix solvers of external libraries, such as PETSc, among other interesting features. The main difference between the *HiGTree/HiGFlow* system and *RheoTool* is that the latter uses a Finite Volume Method (FVM) approach for the numerical simulation of flows. Full details and the theory behind the single-phase flow solvers used in *RheoTool* can be found in [34,35]. Another difference between these two softwares is that the governing equations are solved in a dimensional form in *RheoTool*. Thus, all variables’ units (in SI base units) have to be specified there.

In all the simulations carried out in *RheoTool*, we will use the solver *RheoFoam*, which implements the transient and incompressible Navier-Stokes equations for single-phase flows of Generalized-Newtonian or viscoelastic fluids. In addition, the following techniques will be specified in the *constant* folder and in the *fvSchemes* and *fvSolutions* files: the coupling between stress and velocity was performed using the *Improved Both Sides Diffusion technique* [36]. For the solution of the linear systems resulting from the discretisation of the velocity, the Bi-CGSTAB (BiConjugate Gradient Stabilized) method [37] was used with a DILU (Simplified Diagonal-based Incomplete LU) preconditioner and for the pressure, the conjugated pre-conditioned gradients (PCG) method was used with DIC (Simplified Diagonal-based Incomplete Cholesky) preconditioner.

The following discretisation schemes were used: firstly, we used an Euler implicit scheme for the time derivatives; secondly, all the convective terms were discretised using the CUBISTA method; a Gauss linear scheme was used for the gradient of the pressure and of the velocity and for the divergence terms; finally, the laplacians were discretised using a Gauss linear corrected. All the details of these schemes can be found in [38].

### 3.4. Vorticity-Velocity Code

This code was developed to solve the equations using the Vorticity-Velocity Formulation (*VVF*). The vorticity component in the *z* direction, $\omega_{z}$ can be calculated as:

(54) $$\omega_{z}=\frac{\partial u}{\partial y}-\frac{\partial v}{\partial x},$$

where *u* and *v* are the velocity components in the *x* and *y* directions, respectively (u=(u,v,0)). The equation system to be solved is given by:

(55) $$\frac{\partial \omega_{z}}{\partial t}+\frac{\partial (u\omega_{z})}{\partial x}+\frac{\partial (v\omega_{z})}{\partial y}=\frac{\beta }{Re}\left(\frac{\partial^{2}\omega_{z}}{\partial x^{2}}+\frac{\partial^{2}\omega_{z}}{\partial y^{2}}\right)+\frac{\partial^{2}T_{xx}}{\partial x\partial y}+\frac{\partial^{2}T_{xy}}{\partial y^{2}}-\frac{\partial^{2}T_{xy}}{\partial x^{2}}-\frac{\partial^{2}T_{yy}}{\partial x\partial y},$$

(56) $$\frac{\partial^{2}v}{\partial x^{2}}+\frac{\partial^{2}v}{\partial y^{2}}=-\frac{\partial \omega_{z}}{\partial x},$$

(57) $$\frac{\partial u}{\partial y}+\frac{\partial v}{\partial x}=0,$$

where $T_{xx}$, $T_{xy}$ and $T_{yy}$ are the elastic stress tensor components.

The integration in time (Equation (55)) is carried out by a classical fourth-order Runge-Kutta scheme. The spatial derivatives are discretized by high-order compact finite-difference schemes [39]. After each Runge-Kutta step, the *v* component velocity is calculated by solving the Poisson Equation (56) using a multigrid Full-Approximation Scheme [40]; and the *u* velocity component is updated by the continuity Equation (57). The code is parallelized in the streamwise and wall-normal direction, and the MPI library does the communications. The code adopted here is the same used by [41] for Oldroyd-B studies, changing the non-Newtonian model.

## 4. Results

In the present work, we carried out simulations using the rheological models described in previous sections in two different geometries: 2D channels and planar-contraction 4:1. Firstly, we are interested in simulating shear-banding viscoelastic micellar solutions that obey the VCM model (see Section 2.2). Secondly, we will be simulating materials that exhibit elastoviscoplasticity using the Saramito general equation (see Section 2.4)), which is able to reproduce the behaviour predicted by classical models such as the Oldroyd-B-Bingham, the Oldroyd-B-Herschel-Bulkley and the PTT-Bingham models (both linear and exponential). In addition, our results that will be obtained using the *HiGFlow* software, will then be compared with the solutions from *RheoTool* and from the numerical results derived from the Vorticity-Velocity-Formulation (*VVF*) in order to carry out a code validation process.

In all the simulations performed in the *HiGFlow* system, we used the numerical method that is explained in Section 3. In addition, we use the incremental projection method and a semi-implicit Euler method for temporal-discretisation of the momentum equations, while the viscoelastic and the additional governing equations (for instance, the density number equations of the two species modelled by the VCM model) are discretised using an explicit Euler method. All the details of the interpolation and discretisation schemes used in *RheoTool* were already mentioned in Section 3.3.

### 4.1. Two-Dimensional Channel-Flow

**Figure 5:**
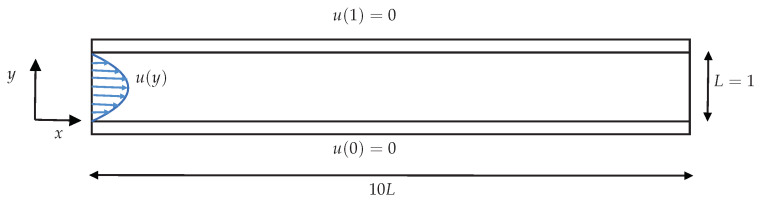
Flow geometry: two-dimensional channel flow.

Here we simply describe the planar channel-flow geometry that is used in *HiGFlow*, *RheoTool* and in the *VVF* approach, see Figure 5. The channel height is *L*, which takes a value of L=1 m, and has an extent of 10L in the x-direction. At the inlet, we set a velocity profile u(y), which in all of the cases will adopt a parabolic shape of the form $u\left(y\right)=U_{0}4y\left(1-y\right)$, where $U_{0}$ is the centreline velocity. At the walls (y=0 and y=1), we have non-slip boundary conditions (u(0)=u(1)=0). Lastly, we set fully developed boundary conditions at the outlet.

### 4.2. The VCM Model

In the present section, we show our simulations in the two-dimensional channel flow using the VCM model (see Section 2.2). To carry out a code verification and validation of our numerical method (see Section 3.2), we will compare directly our results obtained by the *HiGTree/HiGFlow* system with the solutions predicted by *OpenFOAM/RheoTool* and the *VVF* approach. We will start by using an uniform Cartesian mesh with cell sizes Δx=Δy=0.03125 m and with a time-step $\Delta t=1.0\times 10^{-4}$ s.

Besides the boundary conditions for the velocity field mentioned in Section 4.1, we also need to specify the boundary conditions for the tensors (A, B, T) and for the densities ($n_{A}$ and $n_{B}$) and their respective fields in order to initialise the mesh. At t=0, the number densities and stresses are assumed to be initially at equilibrium and thus, we have the following profiles, which can be easily obtained by setting to zero all the time derivatives and the convective terms of the governing Equations (17)–(23):

(58) $$A=I\;B=\frac{n_{B}}{2}I\;T=0$$

(59) $$n_{A}=1\;n_{B}=\sqrt{2\frac{c_{A_{Eq}}}{c_{B_{Eq}}}}.$$

At the walls and at the outlet, we simply set zero gradient boundary conditions for all these quantities.

#### 4.2.1. First Flow Scenario: Low Velocity Values

For our first simulation using the VCM model, we focus on using low velocity values, i.e., $U_{0}=0.125\;m/s$, which is a flow region where we still expect to observe a monotic behaviour in the flow curve (stress vs. shear rate). We will be using parameter values that were fitted to experimental data of a concentrated cetyl pyridinium chloride/sodium salicylate (CPyCl/NaSal) solution, whose rheological parameter values were previously reported in [9,29]: $\lambda_{A}=1.9\;s,\;\lambda_{B}/\lambda_{A}=6.27\times 10^{-4},\;\lambda_{eff}=1\;s$, $D_{A}=D_{B}=1\times 10^{-3}\;L^{2}\;s^{-1},$$C_{A_{Eq}}=0.9,C_{B_{Eq}}=1.4,\chi =0.57$ and $G_{0}=1$ Pa. For our convenience, we choose the following values of density $\rho =10\;\mathrm{Kg}\cdot m^{-3}$ and solvent viscosity $\eta_{s}=0.01\;\mathrm{Pa}\cdot s$. Thus, our dimensionless parameter values (derived in Section 2.3) are:

(60) $$De_{A}=1.9\;De_{B}=1.1913\times 10^{-3}\;\beta =0.01$$

(61) $$Pe_{A}=Pe_{B}=1000\;\chi =0.57\;C_{A_{Eq}}=0.9\;C_{B_{Eq}}=1.4$$

It is important to point out that the solvent-viscosity ratio we use here β=0.01 differs from the value reported experimentally in [9]. The reason we chose to use a higher value of solvent viscosity is mostly to stabilise our numerical method which uses the both-sides diffusion approach, described in Section 2.1. For lower β values, we observed numerical instabilities and therefore, we could not get numerical convergence for this particular model. For similar reasons, we will be particularly focused on simulating VCM fluids with very low values of elasticity number (E≪1).

**Figure 6:**
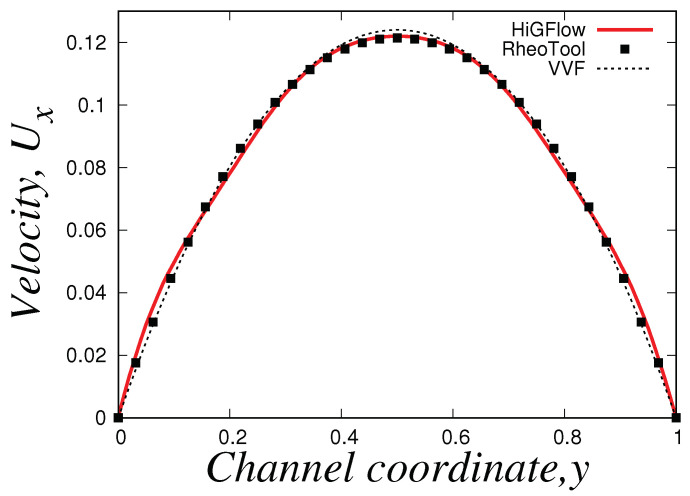
Steady−state velocity profiles $U_{x}$ obtained using *HiGFlow* (red solid line) and the *RheoTool* software (solid black squares) for a VCM fluid with $U_{0}=0.125$ and E=0.1. These results are also compared with the numerical results predicted by the *VVF* approach (dashed line). The VCM model parameter values used are shown in Equations (60) and (61).

We simulate the transient flow of viscoelastic micellar solutions that follow the rheological behaviour of the VCM model, and we will show the fully developed steady-state profiles at x=9.0 using the model parameters shown before for the case E=0.1. In Figure 6, we illustrate the numerical simulations obtained by two CFD software: *HiGFlow* and *RheoTool*, whose solutions are also compared with the results predicted by the *VVF* approach from our in-house code. Although we observe very small differences near the wall and at the centreline, it can be clearly seen that there is a good agreement between the solutions obtained by our three numerical methods.

**Figure 7:**
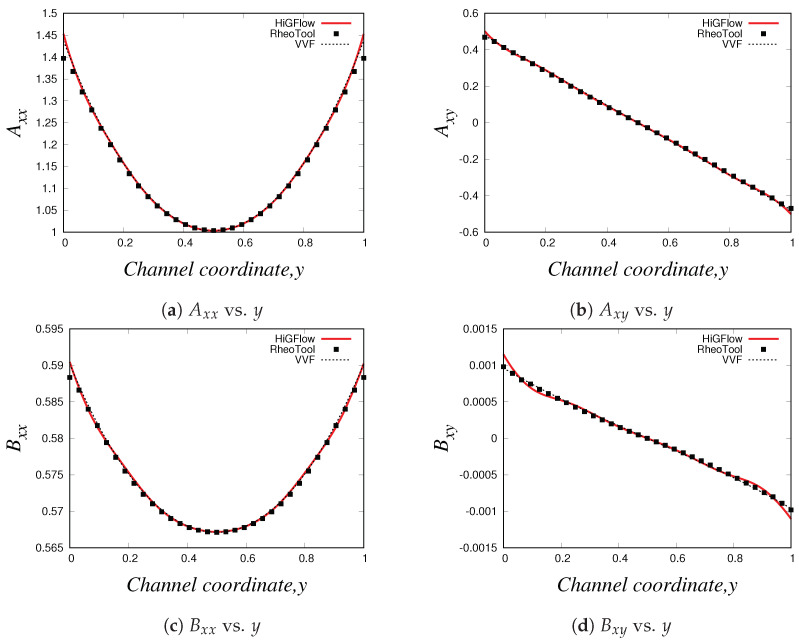
Steady−state profiles of the conformation tensors of the two species (tensors A and B) for a VCM fluid with $U_{0}=0.125$ and E=0.1. (**a**,**b**) profiles for the components of the conformation tensor of specie *A*, $A_{xx}$ and $A_{xy}$; (**c**,**d**) components of the conformation tensor of specie *B*, $B_{xx}$ and $B_{xy}$. The curves in red solid line are the profiles obtained using *HiGFlow*, the solid black squares are the results predicted by *RheoTool* software (solid black squares) and the curves of numerical results obtained by the *VVF* approach are shown as dashed lines. The VCM model parameter values used are shown in Equations (60) and (61).

As described in Section 2.2, the VCM model solves two viscoelastic equations for the conformation tensor of each of the two species, the longer chains (tensor A) and the shorter chains (tensor B). In Figure 7, we show the relevant components of these tensors. Firstly, we have the normal $A_{xx}$ and shear $A_{xy}$ components of the conformation tensor of specie *A*, see Figure 7a,b, where we observe that the first follows a parabolic behaviour while the later is a straight line. On the other hand, Figure 7c,d show the channel profiles for the normal and shear components of the tensor B, whose behaviour is almost identical to the one observed for the profiles of the tensor A. We can notice however that the order of magnitude of the components of the conformation tensor of specie *A* is much more bigger compared to the ones from the conformation tensor of specie *B*, and this is because we are simulating a flow region where the specie *A* (longer chains) dominates, since the flow (low velocity values $U_{0}=0.125$) is not strong enough to cause high breakage rates in the network.

**Figure 8:**
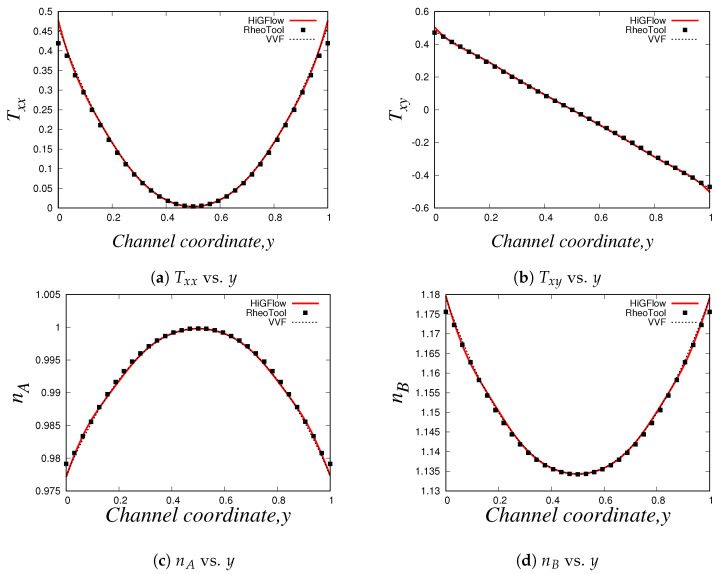
Steady−state profiles of the elastic stress tensor T and density numbers of the species *A* and *B* for a VCM fluid with $U_{0}=0.125$ and E=0.1. (**a**,**b**) profiles for the normal ($T_{xx}$) and shear ($T_{xy}$) stresses of the tensor T; (**c**,**d**) density number of species *A* ($n_{A}$) and *B* ($n_{B}$). The curves in red solid line are the profiles obtained using *HiGFlow*, the solid black squares are the results predicted by *RheoTool* software (solid black squares) and the curves of numerical results obtained by the *VVF* approach are shown as dashed lines. The VCM model parameter values used are shown in Equations (60) and (61).

As shown in Equation (17), the conformation tensors of the two species (A and B) and their respective density numbers ($n_{A}$ and $n_{B}$) have a contribution to the elastic stress tensor T of the network. Figure 8a,b illustrate the relevant components of the tensor T, the normal $T_{xx}$ and the shear $T_{xy}$ stresses, respectively, where it can be seen that the behaviour of the stresses is very similar to the profiles of the conformation tensors reported in Figure 7. The shear stress $T_{xy}$ vs. *y* profile is of particular interest when studying these kind of micellar solutions since it is comparable to the rheological flow curve $T_{xy}$ vs. $\dot{\gamma }$, and this is because the shear rate $\dot{\gamma }$ is proportional to the channel-coordinate *y*. For the present case ($U_{0}=0.125$ and E=0.1), we obtain a monotic curve, which indicates that shear-banding behaviour is not yet observed, and this is due to the low values of velocity, which are not strong enough to cause a high structural destruction of the network. More importantly, we see that there is also a good agreement between the results obtained with the three different methodologies. Small differences between the results can be observed at and near the boundaries, and these differences could be explained by the boundary conditions adopted in each formulation/method. For instance, in *HiGFlow*, the stresses at the wall are determined by the non-slip boundary condition of the velocity. By contrast, in *RheoTool*, we specified zero-gradient and non-slip boundary conditions for the stresses and the velocity, respectively. Despite this, we will show in the following sections that a much better agreement of the results predicted by the three different methodologies at the wall is observed.

Lastly, we have the density number profiles, which are illustrated in Figure 8c,d. Notice how the profile for each specie is different: for specie *A* (longer chains), we observe a maximum value at the centreline, but the concentration of these longer chains decreases continuously as we approach the walls (y=0 and y=1). On the other hand, we observe a minimum value of density number of the shorter chains (specie *B*) at the centreline, but an increase is seen if we move away from y=0.5 and we reach the walls. These results are consistent with the physics of the model, which predicts that the breakage rates are higher at the wall because the viscous dissipation is maximum at y=0 and at y=1. We can also see that the order of magnitude of $n_{A}$ is much greater compared to that of specie *B* ($n_{B}$), and as discussed before, this is because we are simulating a flow region where shear-banding is still not present.

These profiles show that we have an excellent agreement between our results obtained using three different methodologies (especially at the wall, unlike the profiles shown in Section 4.2.1), which manage to capture the basic physics that the VCM model is able to predict. These results also allowed us to carry out a code verification and validation of our numerical method implemented in the *HiGTree/HiGFlow* system described in Section 3.2.

#### 4.2.2. Second Flow Scenario: Shear-Banding Behaviour

It is of our interesting to simulate a flow scenario where shear-banding behaviour is observed, and thus, we will increase the centreline velocity value $U_{0}=10$. We also find convenient to set our elasticity number to E=0.01, which is a smaller value compared to our previous case study (E=0.1), and this is due to the numerical difficulties we faced when simulating VCM fluids at high velocities in both CFD software. Thus, for this, flow case, we set $U_{0}=10\;m/s$, $\rho =100\;\mathrm{Kg}\cdot m^{-3}$ and $\eta_{s}=0.01\;\mathrm{Pa}\cdot s$. In addition, we will be using the same rheological parameter values reported in previous papers [9,29].

**Figure 9:**
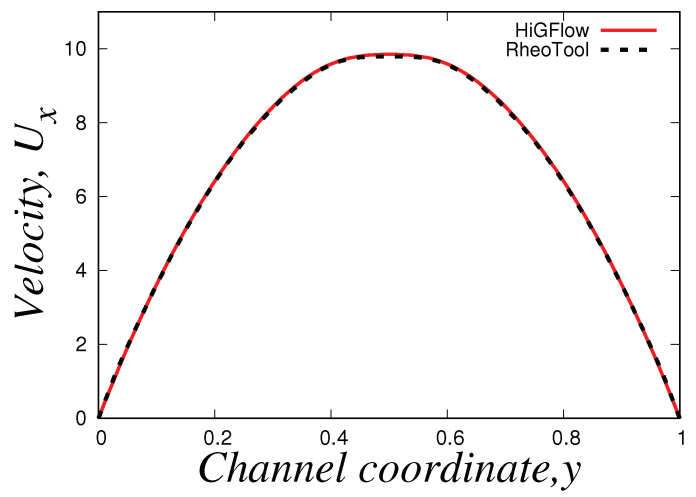
Steady−state velocity profiles $U_{x}$ obtained using *HiGFlow* (red solid line) and the *RheoTool* software (dashed lines) for a VCM fluid with $U_{0}=10$ and E=0.01. The VCM model parameter values used are shown in Equations (60) and (61).

It is also worth mentioning that we could not get good convergence for these parameter values (E=0.01) in our *VVF* in-house code, and thus, we will only report the steady-state profiles obtained using *HiGFlow* and *OpenFOAM/RheoTool* codes. Figure 9 shows the velocity profile in the channel, where it can be easily noticed that there is an excellent agreement in the results predicted by both software. In contrast to the profile observed in the previous section, we can see here that we observe a plug-flow-like profile near the centreline, which indicates us that the low shear-rate region is governed by the longer chains (specie A).

**Figure 10:**
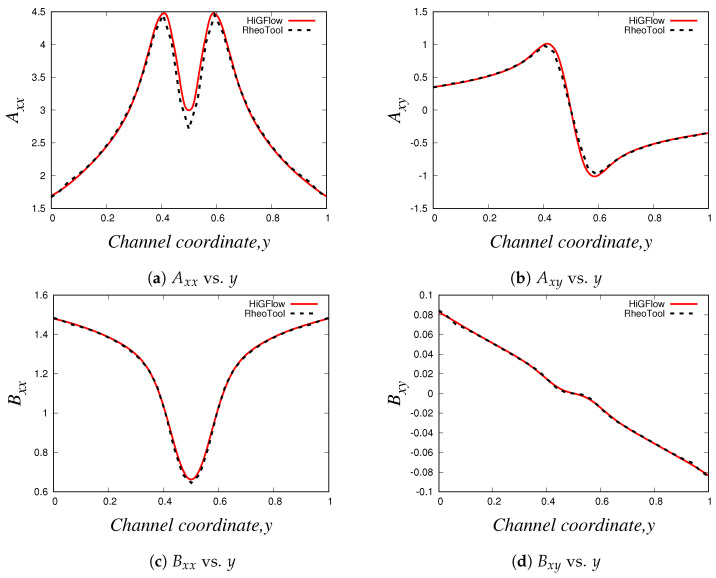
Steady−state profiles of the conformation tensors of the two species (tensors A and B) for a VCM fluid with $U_{0}=10$ and E=0.01. (**a**,**b**) profiles for the components of the conformation tensor of specie *A*, $A_{xx}$ and $A_{xy}$; (**c**,**d**) components of the conformation tensor of specie *B*, $B_{xx}$ and $B_{xy}$. The curves in red solid line are the profiles obtained using *HiGFlow* while the dashed lines represent the results predicted by the *RheoTool*. The VCM model parameter values used are shown in Equations (60) and (61).

The profiles of the normal and shear components of the conformation tensors A and B are shown in Figure 10. Our profiles are in agreement with the results reported by Cromer et al. [29], who carried out numerical simulations of pressure-driven flow of wormlike micellar solutions in rectilinear microchannels (see Figures 6, 7 and 8 in their paper). Notice how the general behaviour of these profiles differ to the one seen in the low-velocity region (see Figure 7). For instance, for the normal component $A_{xx}$ (Figure 10a), here we observe a *V* behaviour at the centreline (where we can also see some slight differences in the results predicted by *HiGFlow* and *RheoTool*), and as we approach the wall, the values of $A_{xx}$ tend to decrease. Another remarkable example is the shear component $A_{xy}$, shown in Figure 10b), where we no longer observe a full linear profile in the whole channel; instead, we observe only a linear behaviour near the centreline; outside of this region, $A_{xy}$ tends to gradually decrease as we are getting closer to the wall (y→0 or y→1). This is a clear indicative that we are starting to observe shear-banding behaviour, since we have a non-monotonic behaviour for the $A_{xy}$ profile.

For the profiles of the components of the tensor B (see Figure 10c,d), we see a perfect agreement between the solutions predicted by both software. Moreover, we can also notice that the order of magnitude of the values of $B_{xx}$ and $B_{xy}$ is greater compared to the previous flow case ($U_{0}=0.125$ and E=0.1), which means that the contribution of species B to the elastic stress tensor T is now more relevant since we have stronger breakage rate caused by the flow ($U_{0}=10$).

**Figure 11:**
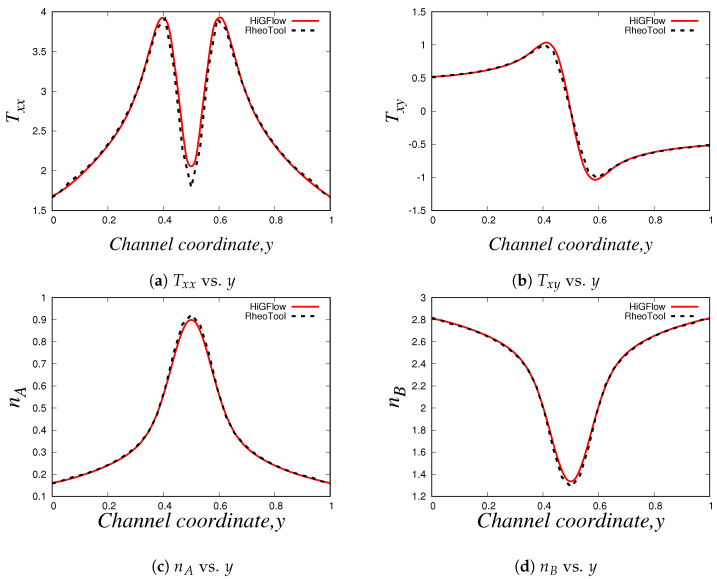
Steady−state profiles of the elastic stress tensor T and density numbers of the species *A* and *B* for a VCM fluid with $U_{0}=10$ and E=0.01. (**a**,**b**) profiles for the normal ($T_{xx}$) and shear ($T_{xy}$) stresses of the tensor T; (**c**,**d**) density number of species *A* ($n_{A}$) and *B* ($n_{B}$). The curves in red solid line are the profiles obtained using *HiGFlow* while the dashed lines are the results predicted by the *RheoTool* software. The VCM model parameter values used are shown in Equations (60) and (61).

We also report the normal $T_{xx}$ and shear $T_{xy}$ components of the tensor T, which can be found in Figure 11a,b, which are indeed very similar to the profiles of the components of the tensor A (see Figure 10a,b). And as we previously discussed in Section 4.2.1, the density number profiles tell us that specie A dominates at the centreline, while we tend to observe a higher number of shorter chains (specie B) near the wall, as shown in Figure 11c,d. Notice how the profiles of $n_{A}$ and $n_{B}$ are less parabolic in contrast to the profiles seen in Figure 11; since we have a stronger flow caused by a higher value of velocity ($U_{0}=10$), the breakage and reformation rates are higher, which leads to a quicker structural destruction of specie A. The rheology and the kinetics of this destruction and reformation of the network is perfectly predicted by *HiGFlow* and *RheoTool*, since our results match nicely.

**Figure 12:**
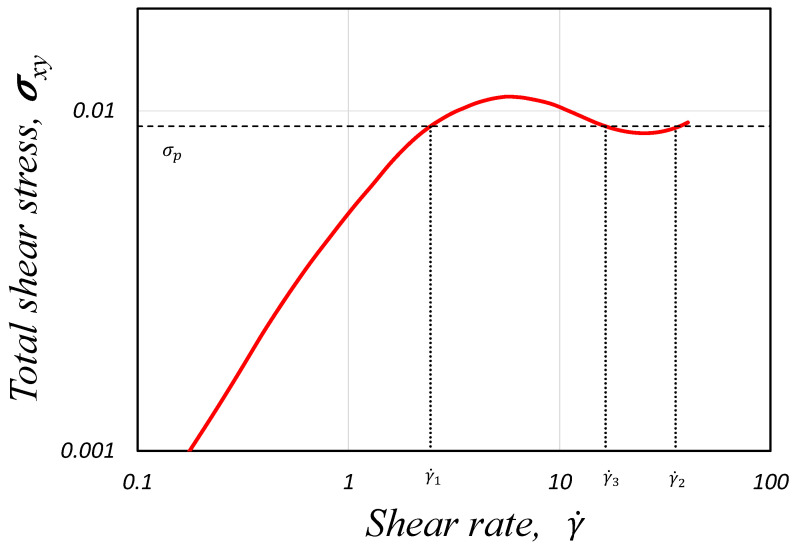
Rheological flow curve ($\sigma_{xy}$ vs. $\dot{\gamma }$) obtained using *HiGFlow* (red solid line) for a VCM fluid with $U_{0}=10$ and E=0.01. The dashed line represents the location of stress plateau $\sigma_{p}$, while the dotted lines indicate the values of the shear rates of the low (${\dot{\gamma }}_{1}$), high (${\dot{\gamma }}_{2}$) and middle (${\dot{\gamma }}_{3}$) band regions that are commonly observed in shear−banded flows.

Lastly, we report the VMC model rheological flow curve (shear stress vs shear rate) that was obtained using our methodology implemented in the *HiGTree/HiGFlow* system. In order to construct the curve, we need the shear rate values $\dot{\gamma }$, which are simply calculated through the gradient of our velocity profile ($\dot{\gamma }=\left|dU_{x}/dy\right|$). Secondly, we will calculate the total shear stress $\sigma_{T}$, which takes into account the solvent contribution and the elastic stress, $\sigma_{T}=S+2\;\left(1+\beta \right)\;E\;D=T+2\;\beta \;E\;D$ and the shear component $\sigma_{xy}$ is then plotted against $\dot{\gamma }$. Our curve is illustrated in Figure 12.

It can be appreciated that the *HiGFlow* system is able to reproduce the typical theoretical non-monotonic flow curve that is observed in flows of micellar solutions (as shown in the introduction, see Figure 1). The dashed horizontal line indicates the location of the stress plateau $\sigma_{p}$, which was calculated using the method of equal areas ($\int_{{\dot{\gamma }}_{1}}^{{\dot{\gamma }}_{3}}\sigma_{xy}\;\dot{\gamma }=\int_{{\dot{\gamma }}_{3}}^{{\dot{\gamma }}_{2}}\sigma_{xy}\;\dot{\gamma }$), where ${\dot{\gamma }}_{1}$ and ${\dot{\gamma }}_{2}$ are the shear-rate value of the bands of fluid with high concentration of specie *A* and specie *B*, respectively, while ${\dot{\gamma }}_{3}$ is the value correspondent to the unstable region of the flow curve (for a more detailed explanation of this rheological curve, see [6]).

#### 4.2.3. Mesh Independence Using the VCM Model

As stated in [25], one of the main advantages of the methodology used by the *HiGTree/HiGFlow* system is the ability to preserve expected order of convergence without any special treatment or geometrical interpolations, which is extremely useful when dealing with non-graded meshes (i.e., meshes that vary abruptly, with large cells being neighbors of very small ones).

In this section, we will carry out simulations of two-dimensional channel-flows using a set of meshes with different cell sizes and refinement levels in order to verify mesh convergence of the results. For these simulations, we will use the same parameter values reported in Section 4.2.2, with a time-step $\Delta t=1.0\times 10^{-4}$.

In Table 1, Table 2 and Table 3, we show the geometric characteristics of the meshes used in our simulations. On one hand, we have the uniform meshes (see Table 1), in which all the cells are equal and have the same cell size (Δx=Δy).

On the other hand, we have the refined meshes, where there are more than one cell sizes. In some flow scenarios it would be ideal to have refined meshes with small cell sizes near the wall to capture properly significant results (since it is the region where we observe a rapid variation of the shear rate and the shear stress) and larger cells near the centreline to reduce overall the computational cost of the simulation since we do not have to simulate the flows using only small cell sizes.

**Figure 13:**
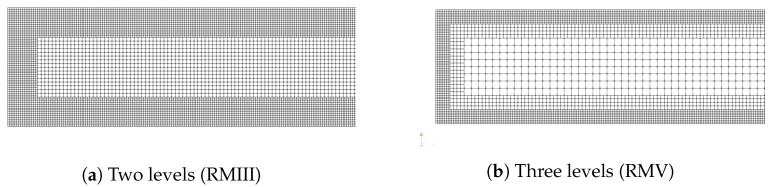
Refined meshes for the two–dimensional channel–flow problem.

In this section, we use meshes with two and three refinement levels, and their geometric characteristics are shown in Table 2 and Table 3. In these tables, we only show the cell size Δx of the large, medium and small cells, but it should be obvious that for each refinement level, Δx=Δy. We also illustrate in Figure 13 some of the meshes to show the refinement near the channel wall.

In order to verify the convergence, we now calculate the errors $L_{1}$, $L_{2}$, $L_{\infty }$. Firstly, we take the mesh MIV as a reference solution, and thus, the errors can be calculated using the following equations:

(62) $$L_{1}=\frac{\sum_{0}^{n}\left|u{\left(i\right)}^{MIV}-u{\left(i\right)}^{*}\right|}{\sum_{0}^{n}\left|u{\left(i\right)}^{*}\right|}\;L_{2}=\sqrt{\frac{\sum_{0}^{n}(u{\left(i\right)}^{MIV}-u{\left(i\right)}^{*}{)}^{2}}{\sum_{0}^{n}{u{\left(i\right)}^{*}}^{2}}}$$

(63) $$L_{\infty }=\frac{max|u{\left(i\right)}^{MIV}-u{\left(i\right)}^{*}|}{max|u{\left(i\right)}^{*}|}$$

where $u{\left(i\right)}^{MIV}$ is the solution in the mesh of reference MIV, $u{\left(i\right)}^{*}$ is the solution of the respective mesh for which the error is being calculated (MI-MIII and RMI-RMV), u(i) is the value of the property (can be velocity, stress, density, etc) in the points i=(x(i),y(i)), where x(i) is a fixed point in the channel x(i)=9 and y(i)=0.01i, with i=0,1,…,100.

Equations (62) and (63) were used to calculate the errors $L_{1},L_{2}$ and $L_{\infty }$ of five quantities predicted by the VCM model: the velocity $U_{x}$, the stresses $T_{xx}$ and $T_{xy}$ and the density numbers of the two species ($n_{A}$ and $n_{B}$). Our results are shown in Table 4, Table 5, Table 6, Table 7 and Table 8. Overall, we can see that all errors of each of our five flow quantities tend to be higher for uniform meshes (MI, MII and MIII) compared to the errors obtained using refined meshes. Subsequently, we can also notice that the errors of the meshes with three levels of refinement (RMIV and RMV) are smaller compared to the meshes with only two levels of refinement (RMI, RMII and RMIII). More interestingly, it can be seen that the errors of the uniform meshes are roughly of the same order of magnitude than the errors obtained in refined meshes that have cells with identical cell sizes Δx; for instance, the uniform mesh MIII which has cell sizes Δx=Δy=0.03125 has error values for the normal stress $T_{xx}$: $L_{1}=4.131\times 10^{-2},L_{2}=1.177\times 10^{-1},L_{\infty }=3.1724\times 10^{-1}$; the error values for refined mesh RMII (which has two levels of refinement with cell sizes Δx=0.0625 near the centre of the channel and Δx=0.03125 near the wall) are $L_{1}=5.380\times 10^{-2},L_{2}=1.675\times 10^{-1},L_{\infty }=3.7873\times 10^{-1}$; lastly, the error values of the refined mesh RMIV that has three levels of refinement whose cells that are close the wall have cell size Δx=0.03125 are: $L_{1}=7.829\times 10^{-2},L_{2}=2.668\times 10^{-1},L_{\infty }=6.1063\times 10^{-1}$. This clearly suggests that instead of simulating uniform meshes with very small cell sizes (which can take plenty of simulation time), we can use refined meshes since we obtain great convergence results thanks to the finite difference method and the meshless interpolation scheme used by the system *HiGTree/HiGFlow* described in Section 3.1.

**Figure 14:**
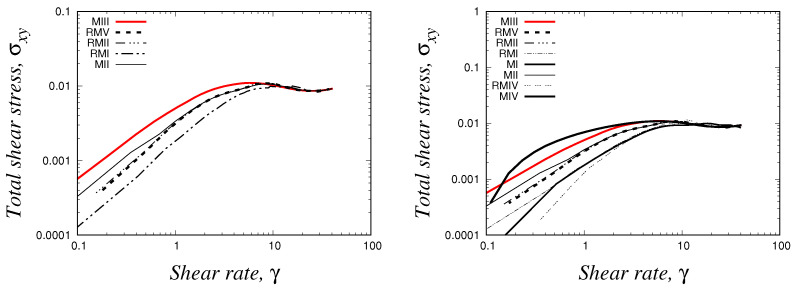
Rheological flow curves ($\sigma_{xy}$ vs. $\dot{\gamma }$) of the VCM model. These curves were obtained by simulating the 2D channel−flow using uniform and refined meshes (see Table 1, Table 2 and Table 3) in the *HiGTree/HiGFlow* system.

Lastly, we show our flow curves ($\sigma_{xy}$ vs. $\dot{\gamma }$), which were obtained using the same meshes reported in Table 1, Table 2 and Table 3. This kind of curve was previously discussed in Section 4.2.2, and the resulting curves of the present analysis can be seen in Figure 14.

On the right, we include all the curves for all the meshes (from MI to RMV), but for better appreciation, we only show the curves of five meshes in the figure of the left, which are the following ones: (1) two uniform meshes (MII and MIII), which are shown as solid lines, (2) two refined meshes with two levels of refinement (RMI and RMII), which are represented by solid-dashed lines and (3) a refined mesh with three levels of refinement (RMV), which is the thick dashed line. It can easily be observed that all the curves predicted exactly the same behaviour seen at intermediate values of shear-rate ($10\ll \dot{\gamma }\ll 100$), which is the region where we observe the shear-banding behaviour. However, some remarkable differences can be appreciated at very low values of shear-rate, and this is due to the different cell sizes that are used in the centreline for the five meshes reported here. As we know, the low $\dot{\gamma }$ values region corresponds to the centreline, which is the location in the channel where specie A dominates, and therefore, if we have meshes with different Δx values, it is expected to obtain shear-stress $\sigma_{xy}$ values with different orders of magnitude. For instance, for the uniform mesh MIII, we obtain a value of $\sigma_{xy}=1.1534\times 10^{-3}$ at $\dot{\gamma }\approx 0.20$, meanwhile we have $\sigma_{xy}=3.7439\times 10^{-4}$ at $\dot{\gamma }\approx 0.20$ for the refined mesh RMV, which has cell sizes Δx=0.0625 at the centreline. In contrast, notice how also the curves predicted using the meshes RMV and RMII overlap each other, and this is because the mesh RMII has also cell sizes Δx=0.0625 at the centre of the channel. These curves also happen to be identical to the curve of the uniform mesh MII, which has Δx=Δy=0.0625. Once again, we conclude here that our methodology used by the *HiGFlow* software in hierarchical grids is able to reproduce flow curves of high interest by the rheology community.

### 4.3. Elastoviscoplastic Fluids: The Saramito Model

The Saramito model [23,24] is used to simulate the rheological behaviour of elastoviscoplastic fluids. Such model combines the viscoplastic and yield stress effects from the Herschel–Bulkley model with the viscoelastic behaviour predicted by models such as the Oldroyd-B and Phan–Thien–Tanner (PTT).

In this section, we will simulate the 2D channel flow using three different methodologies: the Vorticity-Vorticity-Formulation (see Section 3.4), the *OpenFOAM/RheoTool* system that uses the FVM approach (see Section 3.3) and the *HiGTree/HiGFlow* system which uses finite difference methods in tree-based grids (see Section 3.1). The results of our simulations using four different models that can be derived from the general constitutive equation of the Saramito model (Oldroyd-B-Bingham, Oldroyd-B-Herschel–Bulkley, LPTT-Bingham and EPTT-Bingham, see Section 2.5) are shown hereafter.

#### 4.3.1. The Oldroyd-B-Bingham Model

Similarly to the results section of the VCM model, we will use here a simple 2D channel geometry (see Section 4.1), with non-slip boundary conditions at the wall and fully-developed conditions at the outlet for the velocity. At the inlet, we will set a parabolic velocity profile for simplicity.

In order to simulate a flow of the simplest elastoviscoplastic model, the Oldroyd-B-Bingham model (see Equation (40)), we will use the following model parameter values: Re=1.0, De=1.0, ξ=0, β=0.01 and a Bingham number Bi=1.0.

**Figure 15:**
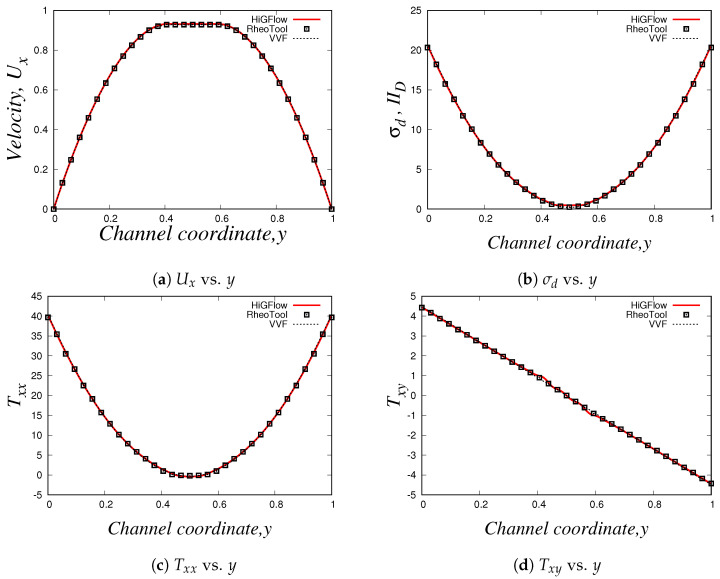
Steady−state profiles of the 2D channel flow simulation of the Oldroyd–B–Bingham model with Re=1.0, De=1.0, ξ=0, β=0.01 and Bi=1.0. The curves in red solid line are the profiles obtained using *HiGFlow*, the black squares are the results predicted by the *RheoTool* software and the curves of numerical results obtained by the *VVF* approach are shown as dashed lines.

Using the three numerical methods described before, and an uniform Cartesian mesh with cell sizes Δx=Δy=0.03125 m and with a time-step $\Delta t=1.0\times 10^{-4}$ s, we obtain the steady-state velocity $U_{x}$, normal and shear stresses ($T_{xx}$ and $T_{xy}$) and the second invariant of the deviatoric stress tensor $\sigma_{d}$ or $II_{D}$ ($\sigma_{d}=\sqrt{(T_{D}:T_{D})/2}\;$) profiles at x=9.0. Our results for the simulation of the Oldroyd-B-Bingham model are illustrated in Figure 15.

As it can be easily seen, there is an excellent agreement between the solutions obtained using *HiGFlow, RheoTool* and the in-house code built with the *VVF* approach, and therefore, our methodology described in Section 3.2 has been successfully implemented and validated in the *HiGTree/HiGFlow* system in order to simulate elastoviscoplastic flows.

The profiles shown above also illustrate the typical behaviour observed in these kind of materials; for instance, in Figure 15a, we have a plug-flow profile for the velocity, where there is solid-like behaviour (or a region of extremely high-viscosity) in the centreline, but a viscous region is observed as we approach the wall. As we know, the yield-stress concept allows us to distinguish between these two regions: if the stress applied is smaller than the yield-stress, we are in the plug-flow region (otherwise we are in the viscous region). But at what exact channel-coordinate *y* we observe the transition from one region to the other one? Figure 15b helps us in answering this question, since we are plotting the second invariant of the deviatoric stress tensor $\sigma_{d}$ (or $II_{D}$). This scalar quantity is used in the constitutive equation reported in Equation (40), where we compare the value of $\sigma_{d}$ with the value of the Bingham number Bi, which is our dimensionless shear stress and Bi=1.0 for our current flow scenario; if $\sigma_{d}<Bi=1.0$, we are in the plug-flow region. It can be easily spotted in our plot that at y≈0.42 (or y≈0.58), the value of $\sigma_{d}$ becomes greater than our Bingham number value Bi=1.0, and thus, we can conclude that the plug-flow region is observed at 0.42≪y≪0.58, and outside this range we observe viscous (or viscoelastic for this case) behaviour.

Figure 15c,d illustrate the profiles of the shear $T_{xy}$ and normal $T_{xx}$ stresses, where we observe their respective typical linear and parabolic profiles of such quantities.

Similarly to our results presented in Section 4.2.3, we also carried out a mesh convergence analysis in *HiGFlow* using the Oldroyd-B-Bingham model: we simulate the 2D channel-flow problem in different meshes (uniform meshes and refined meshes with two and three levels of refinement, see Table 1, Table 2 and Table 3, and we estimate the errors $L_{1}$, $L_{2}$, $L_{\infty }$ using Equations (62) and (63). Our numerical values are reported in Table 9, Table 10, Table 11 and Table 12.

As we can see, the errors tend to be smaller if we use refined meshes with smaller cell sizes, but more interestingly, we are also observing that the order of magnitude of the errors of the uniform meshes (for instance, mesh MIII with Δx=Δy=0.03125) is roughly similar to the errors of the refined meshes that have refinement levels with cell sizes Δx=0.03125 (for instance, meshes RMII and RMIII), which clearly indicate us what we have previously concluded: the finite difference and the meshless interpolations methods in tree-based grids used by the *HiGTree/HiGFlow* allow us to obtain excellent results with good convergence without having to use uniform meshes with very small cell size values.

#### 4.3.2. The Oldroyd-B-Herschel-Bulkley Model

Now we carry out simulations for the 2D channel in our in-house code, *HiGFlow* and in *RheoTool* using the Oldroyd-B-Herschel-Bulkley model, which has an additional parameter: the power-law parameter *n*, see Equation (40). If n=1, we recover the Oldroyd-B-Bingham model, but if n<1, the fluid will exhibit viscoelastic shear-thinning properties if the flow overcomes the yield-stress value. For our simulations, we will use the following parameter values: Re=1.0, De=1.0, ξ=0, β=0.01, Bi=1.0 and n=0.75.

**Figure 16:**
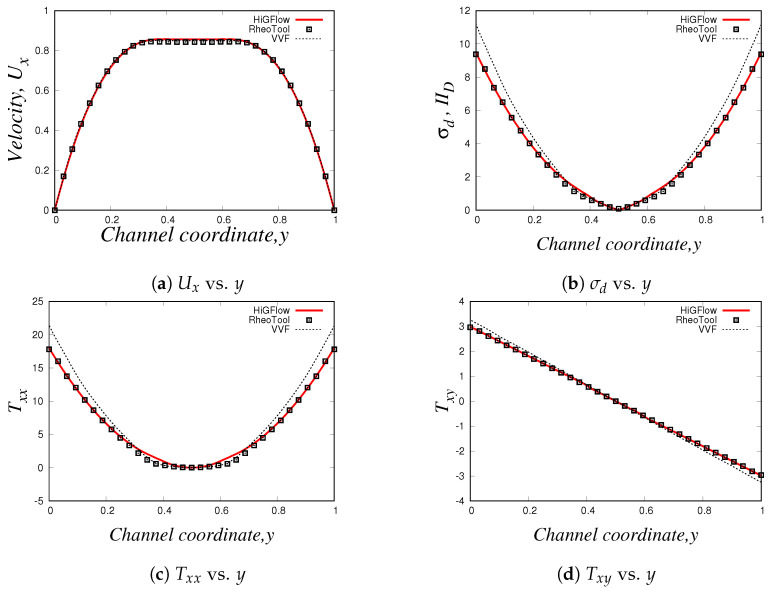
Steady−state profiles of the 2D channel flow simulation of the Oldroyd–B–Herschel–Bulkley model with Re=1.0, De=1.0, ξ=0, β=0.01, Bi=1.0 and n=0.75. The curves in red solid line are the profiles obtained using *HiGFlow*, the solid black squares are the results predicted by the *RheoTool* software and the curves of numerical results obtained by the *VVF* approach are shown as dashed lines.

Our profiles, that were obtained using the three different methodologies in uniform meshes (Δx=Δy=0.03125), are illustrated in Figure 16. Something worth mentioning is that for this model and for the PTT models, we have to use longer meshes L=20 in order to let the flow fully develop. Thus, the profiles we show here are at x=18.0.

As expected, the solutions predicted by the software *HiGFlow* and *RheoTool* are in excellent agreement since the curves overlap with each other in the whole range of the channel coordinate *y*. On the other hand, the numerical results obtained in our in-house code that uses the *VVF* approach are also great compared to the solutions of the CFD software, but we can see that there are some slight differences near the wall.

More importantly, we can notice that although the profiles are roughly identical compared to the profiles of the Oldroyd-B-Bingham model shown in Section 4.3.1, the stress values predicted by the Oldroyd-B-Herschel-Bulkley model at the wall almost decreased by half, which is clearly caused by the effect of the shear-thinning behaviour seen when *n* is smaller than the unit (for this case, n=0.75).

**Figure 17:**
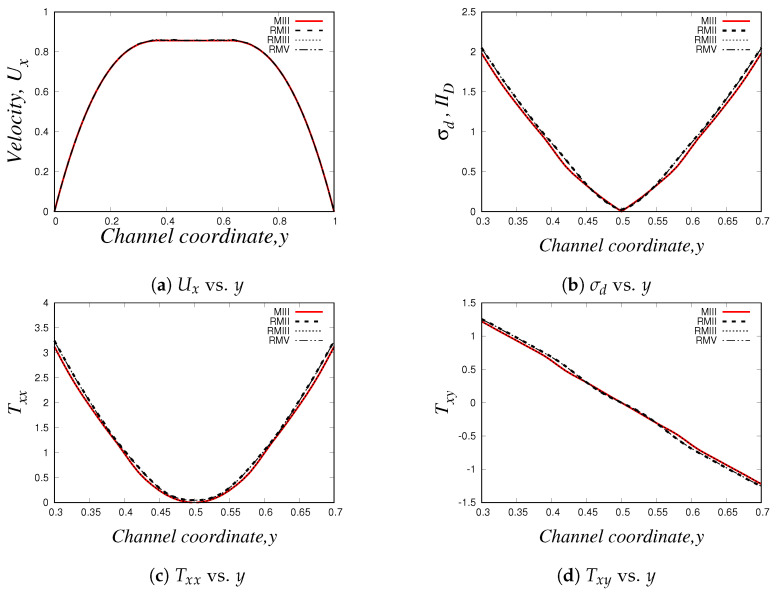
Comparison of the 2D channel–flow profiles using four different meshes in the *HiGTree/HiGFlow* system for the Oldroyd–B–Herschel–Bulkley model with Re=1.0, De=1.0, ξ=0, β=0.01, Bi=1.0 and n=0.75. The meshes used here are: an uniform mesh MIII (red solid line), meshes with two refinement levels, RMII and RMIII (thick and thin dashed lines) and a mesh with three refinement levels RMV (thin solid-dash line). See Table 1, Table 2 and Table 3 for more specific details of the meshes.

We also simulated an Oldroyd-B-Herschel-Bulkley fluid using uniform and refined meshes, and the results were compared, as shown in Figure 17. It is evident that the profiles obtained using different meshes (MIII, RMII, RMIII and RMV, see Table 1, Table 2 and Table 3 are almost identical. However, we take a closer inspection and we show the profiles in the range 0.3<y<0.7 in order to demonstrate that we observe very few differences near the centreline, specially between the solutions of the uniform mesh MIII (Δx=0.03125) and the refined mesh with two levels of refinement RMIII with respect to the meshes RMII and RMV, whose cells near the centreline have a greater cell size value (Δx=0.0625). Once again, this clearly shows that we obtain very decent results by using refined meshes, which take less computational time compared to the simulations obtained using uniform meshes with very small cell size values.

#### 4.3.3. The Linear- and Exponential-PTT-Bingham Models

In this section we will show our numerical results using two variations of the PTT-Bingham models: the linear-PTT-Bingham (LPTT-Bingham) and the exponential-PTT-Bingham (EPTT-Bingham), where a new model parameter ϵ is introduced, which is the extensibility parameter. The parameter values for our simulations are: Re=1.0, De=1.0, ξ=0, β=0.01, Bi=1.0 and ϵ=0.1. The profiles obtained using *HiGFlow, RheoTool* and our in-house code for an uniform mesh with L=20 and with cell sizes Δx=Δy=0.03125 are illustrated below.

**Figure 18:**
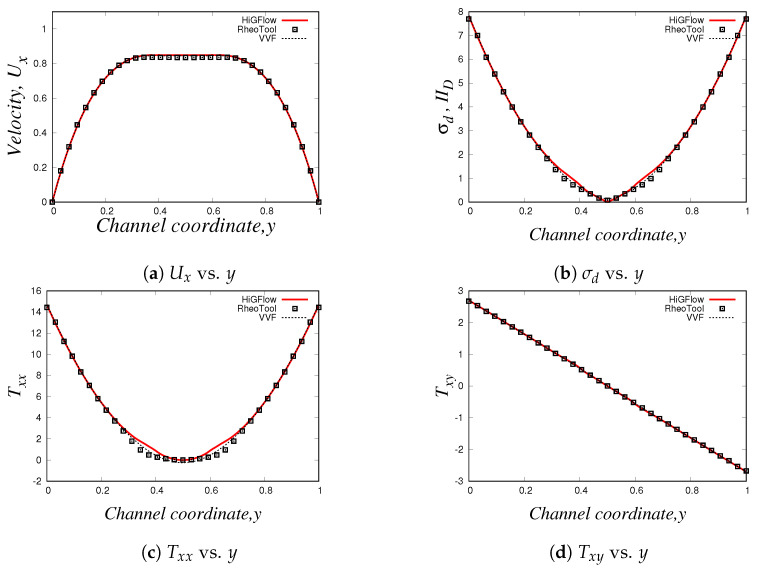
Steady–state profiles of the 2D channel–flow simulation of the LPTT–Bingham model with Re=1.0, De=1.0, ξ=0, β=0.01, Bi=1.0 and ϵ=0.1. The curves in red solid line are the profiles obtained using *HiGFlow*, the solid black squares are the results predicted by the *RheoTool* software and the curves of numerical results obtained by the *VVF* approach are shown as dashed lines.

**Figure 19:**
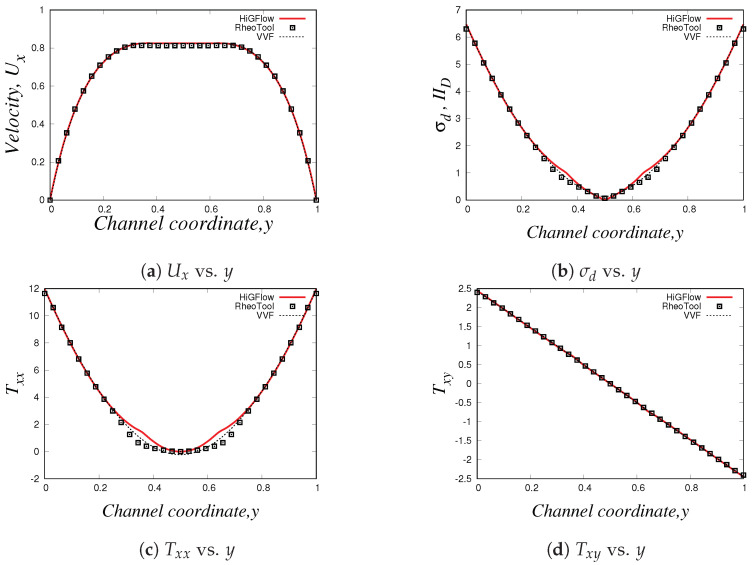
Steady–state profiles of the 2D channel flow simulation of the EPTT–Bingham model with Re=1.0, De=1.0, ξ=0, β=0.01, Bi=1.0 and ϵ=0.1. The curves in red solid line are the profiles obtained using *HiGFlow*, the solid black squares are the results predicted by the *RheoTool* software and the curves of numerical results obtained by the *VVF* approach are shown as dashed lines.

The profiles for the LPTT-Bingham model can be found in Figure 18, while the profiles for the EPTT-Bingham model are in Figure 19. It is not surprising to see that for both models we also obtain a plug-flow velocity profile near the centreline, as well as a parabolic-like and linear profiles for $T_{xx}$ and $T_{xy}$, respectively. We can also see that overall, there is a good agreement between the numerical results predicted by our three methodologies. The region where we notice some differences between our results is in the centreline for the normal stress $T_{xx}$ (see Figure 18c and Figure 19c).

**Figure 20:**
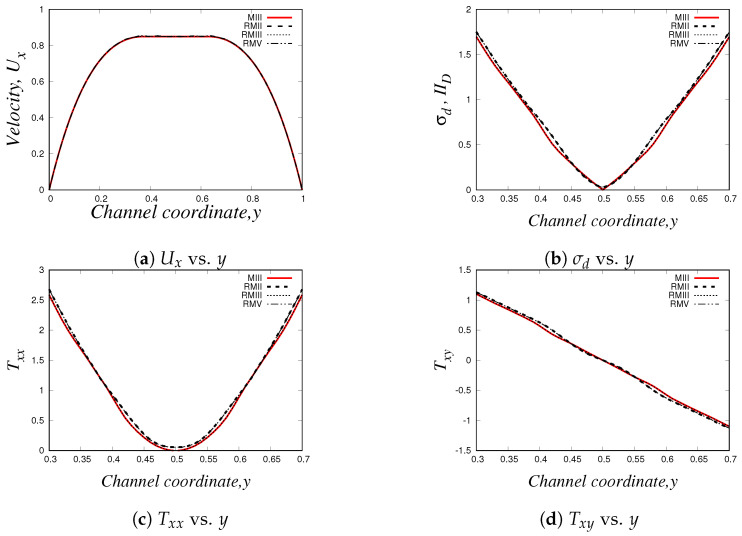
Comparison of the 2D channel–flow profiles using four different meshes in the *HiGTree/HiGFlow* system for the LPTT–Bingham model with Re=1.0, De=1.0, ξ=0, β=0.01, Bi=1.0 and ϵ=0.1. The meshes used here are: an uniform mesh MIII (red solid line), meshes with two refinement levels, RMII and RMIII (thick and thin dashed lines) and a mesh with three refinement levels RMV (thin solid-dash line). See Table 1, Table 2 and Table 3 for more specific details of the meshes.

**Figure 21:**
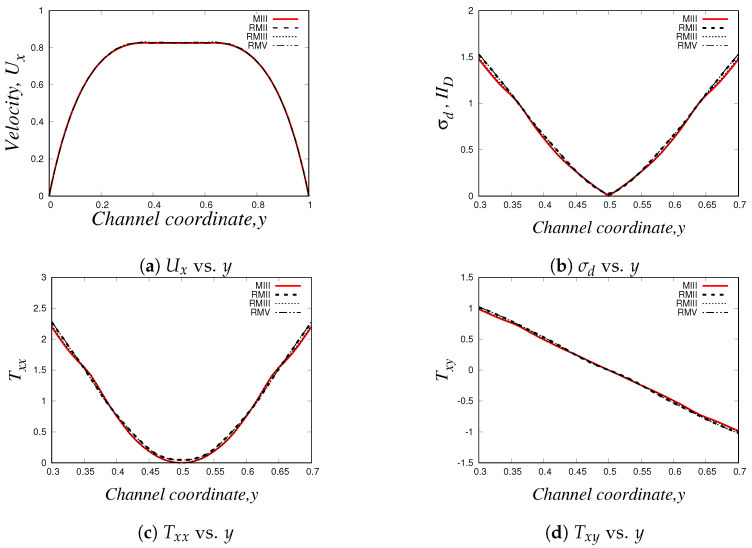
Comparison of the 2D channel–flow profiles using four different meshes in the *HiGTree/HiGFlow* system for the EPTT–Bingham model with Re=1.0, De=1.0, ξ=0, β=0.01, Bi=1.0 and ϵ=0.1. The meshes used here are: an uniform mesh MIII (red solid line), meshes with two refinement levels, RMII and RMIII (thick and thin dashed lines) and a mesh with three refinement levels RMV (thin solid-dash line). See Table 1, Table 2 and Table 3 for more specific details of the meshes.

Similarly to what we showed in the previous section, we also carried out simulations of these models in different meshes (MIII, RMII, RMIII and RMV), and the results are illustrated in Figure 20 and Figure 21. We can also see that although the profiles almost overlap with each other in the whole range of the channel-coordinate, we observe some deviation in the numerical results near the centreline, especially between the uniform mesh MIII and meshes RMII and RMV, which is due to different cell sizes of the meshes located in the centreline, as discussed before. Our results here are in agreement with the mesh convergence results shown in Section 4.2.3, where we stated that the finite difference method in tree-based grids methodology used by *HiGFlow* provides us with good convergence results.

**Figure 22:**
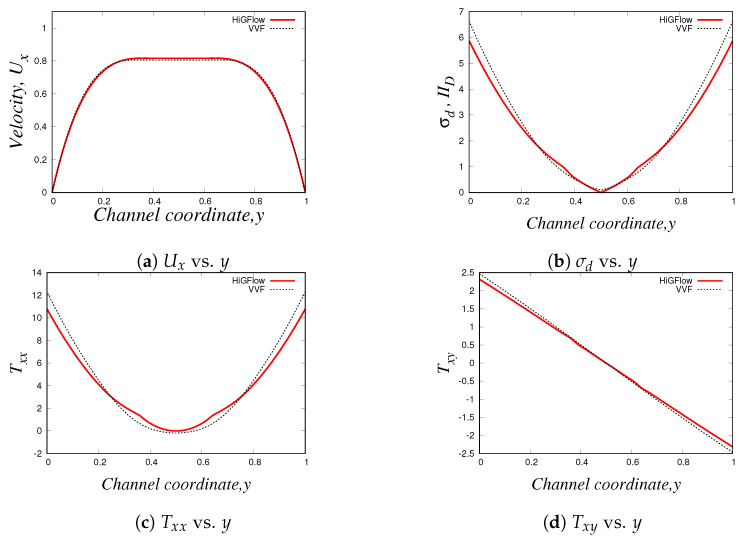
2D channel flow simulation of an elastoviscoplastic fluid that couples the linear–PTT viscoelastic behaviour with the Herschel–Bulkley model. The following parameter values were used: Re=1.0, De=1.0, ξ=0, β=0.01, Bi=1.0ϵ=0.1 and n=0.75. The solid ref line is the solution predicted by *HiGFlow*, while the dashed line represents the numerical results obtained in our in-house code that uses the *VVF*.

Lastly, we wish to conclude the 2D channel flow section by showing results of one last simulation of an elastoviscoplastic fluid that can exhibit a different behaviour. Thanks to the Saramito general model (see Equation (26)), we can choose what kind of behaviour to study by specifying the function f(τ), and thus, we carried out a simulation of a fluid with viscoelastic properties modelled by the linear-PTT model coupled with plasticity behaviour of the Herschel-Bulkley model. The flow parameter values to be used are: Re=1.0, De=1.0, ξ=0, β=0.01, Bi=1.0, ϵ=0.1 and n=0.75. It is worth mentioning that the simulation of a LPTT-Herschel-Bulkley fluid cannot currently be carried out in *OpenFOAM/RheoTool*, since the regularisation of PTT models is not allowed if n≠1 (see [28]). Therefore, we only show the numerical results predicted by *HiGFlow* and the *VVF* approach, see Figure 22.

If we compare these results with the profiles obtained for the LPTT-Bingham model (see Figure 18), the values near the wall of the components of the stress tensor ($T_{xx}$ and $T_{xy}$) and of the second invariant of the deviatoric stress tensor $\sigma_{d}$ tend to be smaller for the LPTT-Herschel-Bulkley model, which is a clear effect of the index value n=0.75. Notice how also both methodologies predict the same behaviour for all curves, but there are some regions (specially close to the walls) in the channel when the solutions differ, which is something we also observe in our results for the Oldroyd-Herschel-Bulkley model (see Figure 16).

### 4.4. The Planar 4:1 Contraction Flow

**Figure 23:**
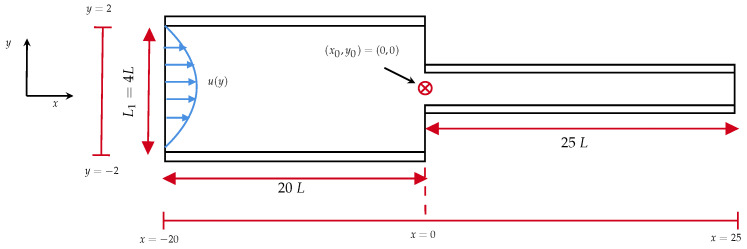
Domain representation: the planar 4:1 contraction geometry.

In the previous sections, we showed our numerical results in a planar channel geometry, but here we are interested in simulating VCM and elastoviscoplastic fluids in a more complex geometry: the planar-contraction 4:1 (see a sketch of the geometry in Figure 23), which offers a mix of shear and extensional deformation near the contraction region and where secondary flows might exist. This kind of flow is a suitable benchmark problem for the evaluation of new models or numerical methods.

Some of the main characteristics of this geometry are: the width of the downstream channel is denoted by a characteristic height L=1 m, and, as required by the problem, the width $L_{2}$ of the upstream channel is $L_{2}=4L$. In addition, the inlet and outlet effects can be neglected since the downstream and upstream lengths are 25L and 20L, respectively. From our sketch of the domain, we can see that the origin $\left(x_{0},y_{0}\right)=\left(0,0\right)$ is located where the contraction begins, exactly at the centreline of the downstream channel.

**Figure 24:**
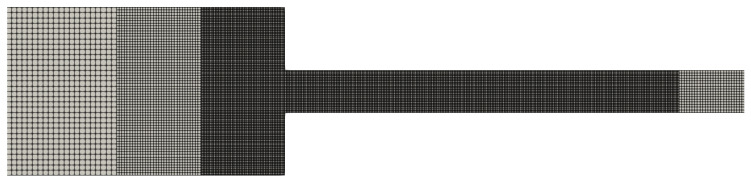
Refined mesh used in *HiGFlow* for the contraction 4:1 flow problem with 3 levels of refinement.

**Figure 25:**
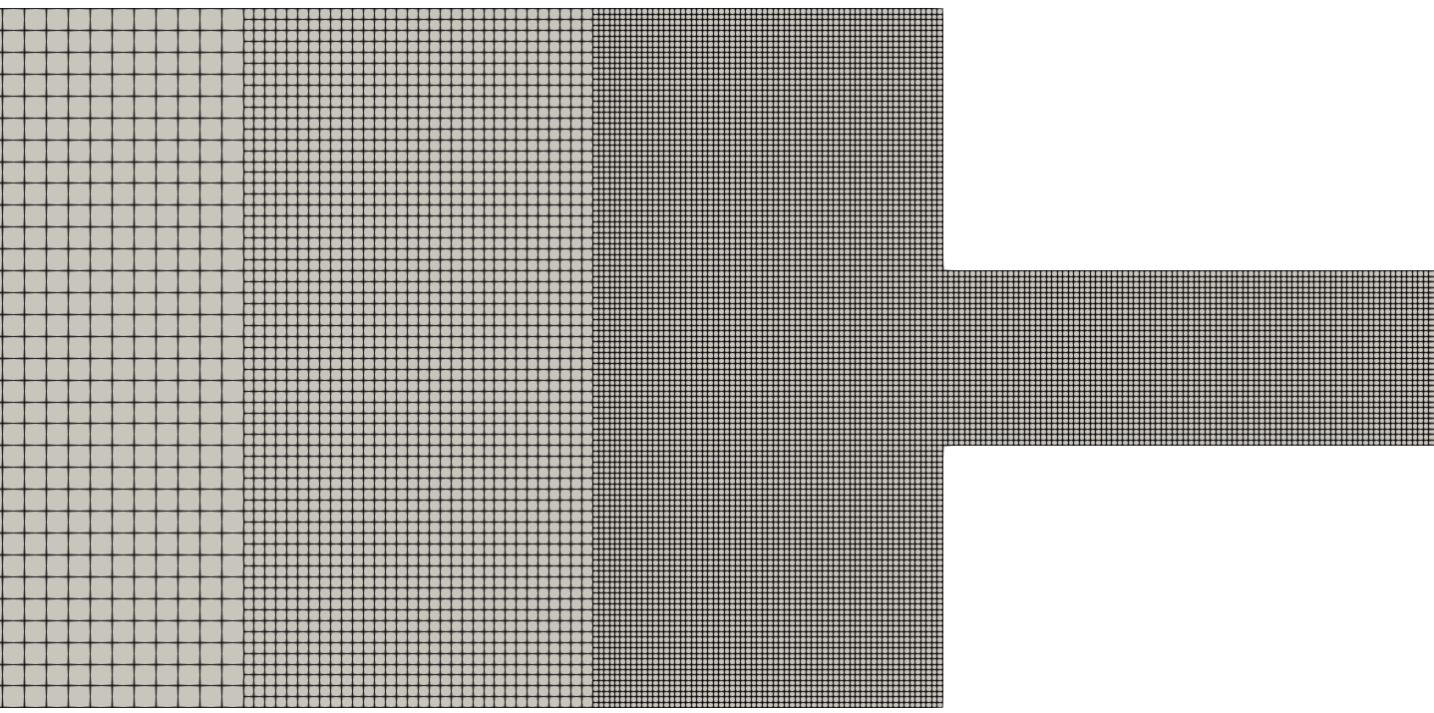
Refined mesh RM used in *HiGFlow* near the contraction region.

In all simulations for the contraction problem carried out in *HiGFlow*, we use mesh RM, which is displayed in Figure 24. This mesh has three levels of refinement, with the most refined part near the contraction region, with cell sizes Δx=Δy=0.03125 (see Figure 25 for a better appreciation). The second level of refinement has cell sizes Δx=Δy=0.0625 and the third one (whose cells mostly cover the inlet and outlet regions) Δx=Δy=0.125. We will also use a uniform mesh UM with Δx=Δy=0.0625 in order to check the convergence of the solutions and to compare results between meshes UM and RM.

**Figure 26:**
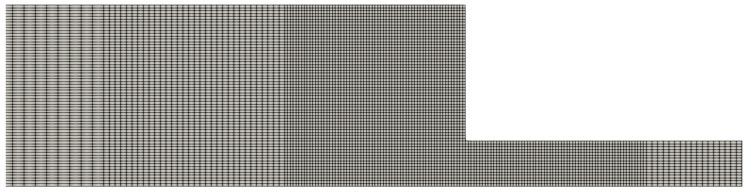
Refined mesh RMRT used in *OpenFoam/RheoTool* (3 refinement levels).

On the other hand, we show the mesh RMRT used in *RheoTool* (see Figure 26). It is important to point out that this software solves the governing equations using the finite volume approach, so it is necessary to provide a three-dimensional mesh. This mesh was constructed in OpenFOAM and was adapted in order to have meshes with similar geometric conditions (and as close as possible) to the meshes used in *HiGFlow* (Figure 24 and Figure 25). As it can be seen, in *RheoTool* we only simulate half of the domain since the flow is symmetric. In addition, we can notice that the upstream and downstream regions of the contraction geometry have volumes with exactly the same dimensions of the meshes used in *HiGFlow*. More details of this mesh can be found in our previous work [27]. Finally, in all the simulations of the contraction problem we use the same time-step value $\Delta t=1.0\times 10^{-4}$.

Lastly, it is important to mention that unlike the 2D-channel flow section, here we only focus on performing simulations using CFD software (*HiGFlow* and *RheoTool*), since our in-house code with the Vorticity-Vorticity-Formulation is only designed to solve simple geometries (see Section 3.4).

#### 4.4.1. The VCM Model

In this section, we will show results of our simulations of a VCM fluid in the planar-contraction 4:1 flow problem using the meshes UM, RM and RMRT described in Section 4.4. Once again, we will compare the solutions from *HiGFlow* with the numerical results obtained from *RheoTool*. To our knowledge, the results we are about to present are the first computational simulations carried out in the planar-contraction 4:1 using the VCM model.

We also focus on simulating flows with low velocity values ($U_{0}=0.125\;m/s$), where $U_{0}$ is the centreline velocity of the small channel, since we found numerical instabilities in our simulations for high velocity flows ($U_{0}\gg 1$). Therefore, our selected parameter values are the same ones reported in Section 4.2.1, which are parameters fitted to experimental data of a concentrated cetyl pyridinium chloride/sodium salicylate (CPyCl/NaSal) solution [9,29]: $\lambda_{A}=1.9\;s,\;\lambda_{B}/\lambda_{A}=6.27\times 10^{-4},\;\lambda_{eff}=1\;s$, $D_{A}=D_{B}=1\times 10^{-3}\;L^{2}\;s^{-1},C_{A_{Eq}}=0.9,C_{B_{Eq}}=1.4,\chi =0.57$ and $G_{0}=1$ Pa. For our convenience, we set $\rho =10\;\mathrm{Kg}\cdot m^{-3}$ and $\eta_{s}=0.01\;\mathrm{Pa}\cdot s$. Thus, our dimensionless parameter values (derived in Section 2.3): E=0.01, $De_{A}=1.9,De_{B}=1.1913,\beta =0.01,Pe_{A}=Pe_{B}=1000,\chi =0.57,C_{A_{Eq}}=0.9$ and $C_{B_{Eq}}=1.4$.

We also initialise the densities, tensors and velocity fields using the same values reported in Section 4.2. We set a parabolic profile of the form $U\left(y\right)=U_{0}\left(1/4\right)\left(2-y\right)\left(2+y\right)$ at the inlet, fully developed flow conditions at the outlet, and non-slip conditions for the velocity and zero-gradient boundary conditions for the other fields at the walls.

**Figure 27:**
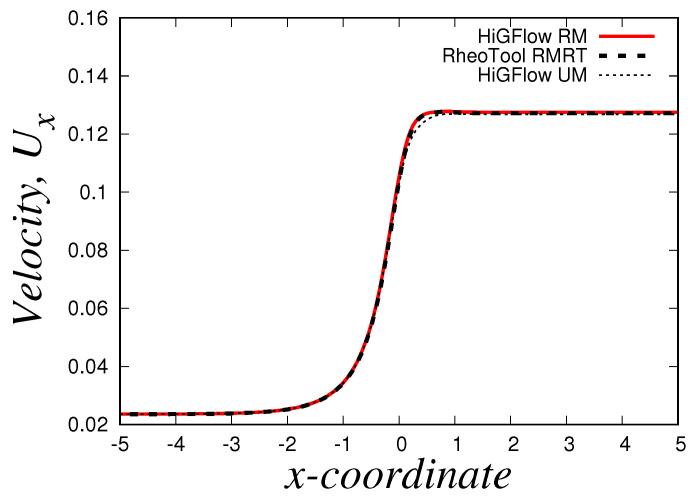
Planar−contraction 4:1 steady-state velocity profiles $U_{x}$ obtained using *HiGFlow* (refined mesh RM in red solid line and uniform mesh UM in dotted line) and the *RheoTool* software (dashed line, with mesh RMRT) for a VCM fluid with $U_{0}=0.125$ and E=0.01.

We start by illustrating the centreline axial velocity profiles near the contraction region (y=0 and −5<x<5) shown in Figure 27. Three curves can be found: two of them correspond to the numerical solutions predicted by *HiGFlow*, one obtained using the refined mesh RM (red solid line) and the black dotted line correspond to the uniform mesh UM. On the other hand, we have the black dashed line for the *RheoTool* results obtained using the refined mesh RMRT described in Figure 26. For this figure, we see the typical velocity profile seen in this geometry: the velocity increases as we approach the contraction region (x=0 and y=0) and it is followed by an overshoot behaviour. After this, the velocity reaches the steady-state centreline velocity of the downstream channel. It is clearly evident that there is an excellent agreement in the solutions obtained by *RheoTool* and *HiGFlow*.

**Figure 28:**
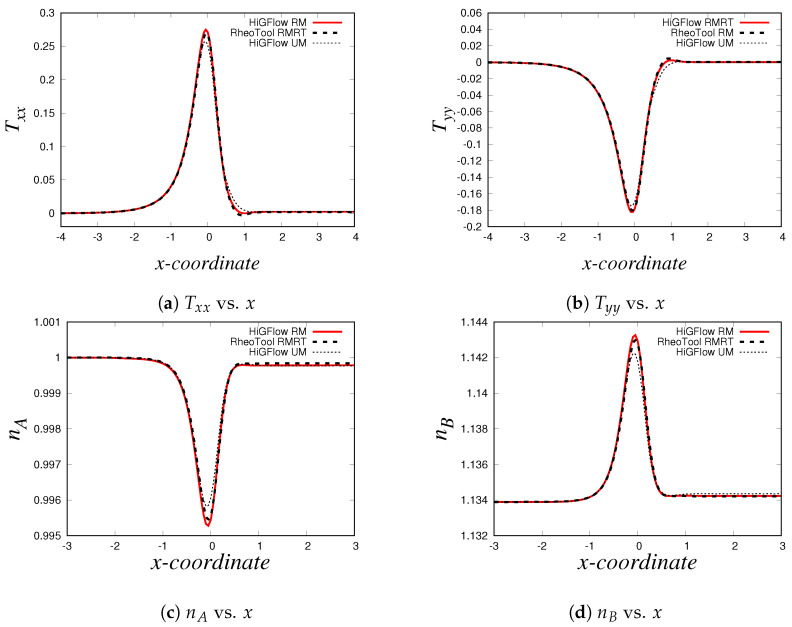
Planar–contraction 4:1 centreline axial profiles of the normal components of the elastic stress tensor ($T_{xx}$ and $T_{yy}$) and the density numbers of the two species ($n_{A}$ and $n_{B}$) of a VCM fluid. The results obtained in *HiGFlow* are the red solid line (refined mesh RM) and the dotted line (uniform mesh UM), while the predictions by *RheoTool* are represented by the dashed line curve (mesh RMRT).

The centreline axial profiles of the other fields predicted by the VCM model (elastic stress tensor T and density numbers of the two species, $n_{A}$ and $n_{B}$) can be found in Figure 28, where we can notice that both *RheoTool* and our methodology implemented in *HiGFlow* (both uniform and refined meshes) are able to reproduce the expected behaviour in contraction 4:1 flows and the solutions of these CFD software that use different approaches (FVM and finite-differences) are in excellent agreement.

We start by describing the profile for the normal stress $T_{xx}$, which can be seen in Figure 28a. Our simulations describe also the common behaviour adopted by $T_{xx}$ in this geometry, where we observe a continuous increase of the normal stress until it reaches a maximum value at the centreline (x=0, y=0). After this point, $T_{xx}$ decays and reaches a zero value very far away from the contraction region (x≫5).

Unlike the 2D-channel flow simulation of previous sections, here we report the normal stress $T_{yy}$, which is a quantity that becomes more relevant in planar-contraction flows, which is found in Figure 28b. As it can be seen, the behaviour of $T_{yy}$ is the complete opposite of $T_{xx}$: $T_{yy}$ has always negative values, and as the fluid enters the contraction region (which is where the extensional flow is extremely relevant), the stress decreases and reaches a minimum, and then it gradually increases until $T_{yy}$ becomes zero.

On the other hand, the profiles of the density number of the longer chains ($n_{A}$) and of the shorter chains ($n_{B}$) can be found in Figure 28c,d, respectively. Their behaviour is very similar to that of the normal stresses: the concentration of both chains is constant ($n_{A}=1$ and $n_{B}=\sqrt{2C_{A_{Eq}}/C_{B_{Eq}}}=1.13389$) until the fluid enters the contraction region zone (−1<x<1), where the extensional flows causes a decrease on the concentration of longer chains (specie A), while simultaneously the density number of the shorter chains (specie B) is increasing. More interestingly, a minimum value of $n_{A}$ and a maximum value of $n_{B}$ are observed exactly at the origin (x=0, y=0). Very far away from the contraction region (x≫5), the density numbers recover its initial values ($n_{A}=1$ and $n_{B}=1.13389$).

**Figure 29:**
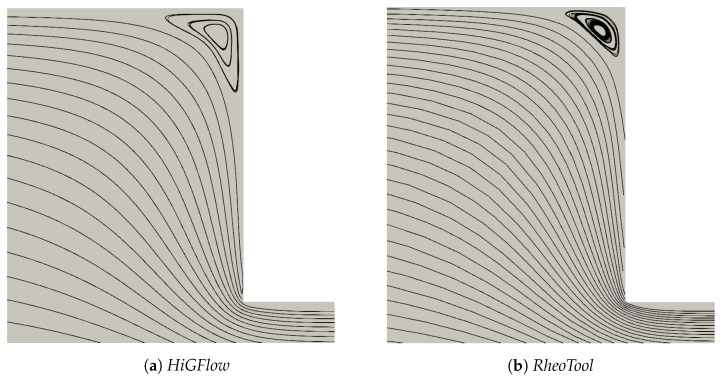
Comparison of the streamlines of the planar–contraction 4:1 flow problem of a VCM fluid.

As reported in the literature, the elastic forces in viscoelastic fluids can generate the formation of a corner vortex near the contraction region [34,42]. Therefore, we wish to show the streamlines obtained both in *RheoTool* and *HiGFlow* near the contraction region, which can be found in Figure 29. All these streamlines were calculated in Paraview, and we used exactly the same resolution parameters. It can be clearly seen that both methodologies (FVM in *RheoTool* and finite-differences in tree-based grids in *HiGFlow*) are able to predict the formation of a corner vortex. However, we can notice that the size of the vortexes differ from each other; for instance, the value of the dimensionless vortex size (horizontal lentgh) $X_{R}$ predicted by *HiGFlow* is $X_{R}=0.482733$, while *RheoTool* predicts a value of $X_{R}=0.313078$, showing a 35% percentage difference between the values.

#### 4.4.2. The Saramito Model

In this last subsection, we will show numerical results from our simulations using the Saramito model in the planar-contraction 4:1 flow problem with meshes UM, RM and RMRT. As previously discussed, the general equation of Saramito allows the user to choose the elastoplastic behaviour to be simulated. Thus, we will focus on simulating a fluid that obeys the Oldroyd-B-Herschel-Bulkley model and another fluid that follows the linear-PTT-Bingham behaviour. The main reason we chose to use these models is that they avoid the possible infinite Oldroyd-B elongational viscosity that is observed in extensional flows of Oldroyd-B fluids for De>0.5.

**Figure 30:**
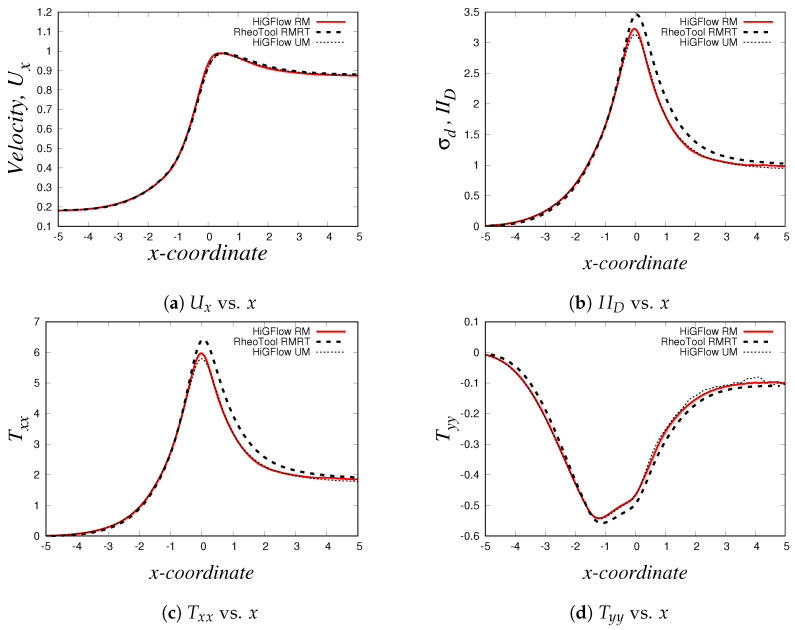
Planar–contraction 4:1 centreline axial profiles of a Oldroyd-B-Herschel-Bulkley fluid. The results obtained in *HiGFlow* are the red solid line (refined mesh RM) and the dotted line (uniform mesh UM), while the predictions by *RheoTool* are represented by the dashed line curve (mesh RMRT).

For the Oldroyd-B-Herschel-Bulkley fluid, we use the following parameter values: Re=1.0, De=1.0, ξ=0, β=0.01, Bi=1.0 and n=0.75. The centreline axial profiles near the contraction region (y=0 and −5<x<5) for this case can be found in Figure 30. Overall, it can be seen that both *HiGFlow* and *RheoTool* predict the same phenomena, and the curves of the numerical results are almost roughly the same, with some small differences in the numerical values seen in the contraction region (0<x<2).

**Figure 31:**
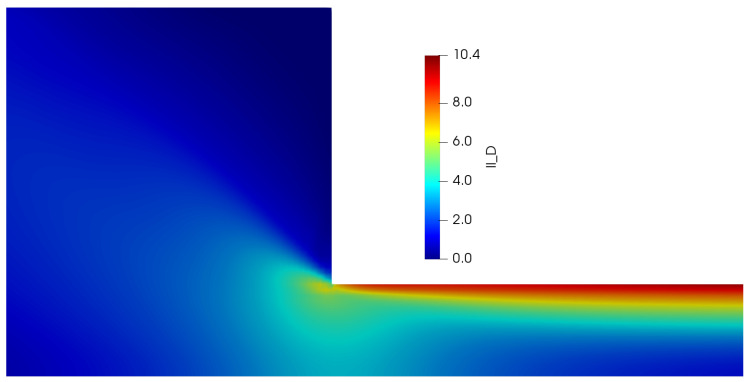
Heat map of the second invariant of the deviatoric stress tensor $\sigma_{d}$ ($II_{D}$) of a Oldroyd–B–Herschel-Bulkley fluid in the planar-contraction 4:1.

For the velocity profile (see Figure 30a), we have the characteristic overshoot that is seen in this planar-contraction geometry. Figure 30b shows the second invariant of the deviatoric stress tensor $\sigma_{d}$, which provides us with interesting information about the flow: before the fluid enter the contraction region (x<−2), it behaves as a rigid solid in the centreline since $\sigma_{d}<Bi=1$; as soon as it enters in this region, we start to observe viscoelastic behaviour and $\sigma_{d}$ reaches a maximum value at x=0. As the fluid leaves the contraction, $\sigma_{d}$ begins to decay gradually until the fluid starts to behave again as a rigid solid at x≫2 (this range is not shown here but see Figure 31 for better visualisation).

On the other hand, we show the normal components of the elastic stress tensor, which are illustrated in Figure 30c,d. These curves are also in agreement with our previous figure, since we observe that the elastic stresses start to become more relevant when the fluid approaches the contraction region (−2<x<2).

In contrast to the flows of PTT fluids or any other viscoelastic fluids without yield stress (see also our results from Section 4.4.1), where we observe that the elastic forces lead to the generation of corner vortexes, we do not observe such vortexes in our simulations of an elastoviscoplastic fluid modelled through the Oldroyd-B-Herschel-Bulkley model. The lack of vortexes can be better explained using Figure 31, where we show a heat-map of the second invariant of the deviatoric stress tensor $\sigma_{d}$ ($II_{D}$). As we can notice, the values of $\sigma_{d}$ in the corner are very small (royal blue colour) and therefore, they do not overcome the value of our Bingham number Bi=1.0, which means that the fluid will behave as a rigid solid in the corner.

**Figure 32:**
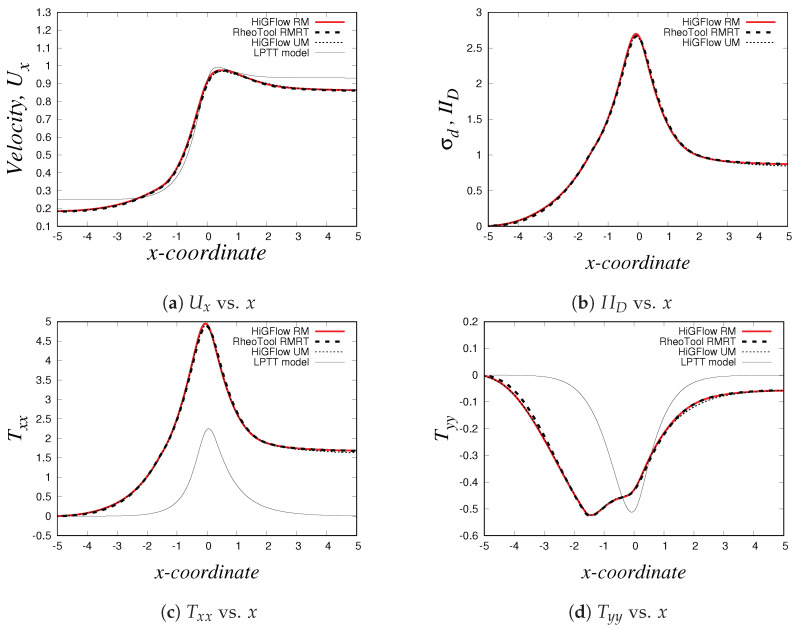
Planar–contraction 4:1 centreline axial profiles of a LPTT–Bingham fluid. The results obtained in *HiGFlow* are the red solid line (refined mesh RM) and the dotted line (uniform mesh UM), while the predictions by *RheoTool* are represented by the dashed line curve (mesh RMRT). The velocity and normal stress profiles of a standard LPTT fluid without yield stress are also included as reference as thin solid lines.

For the last simulation, we use the following parameter values for our LPTT-Bingham fluid: Re=1.0, De=1.0, ξ=0, β=0.01, Bi=1.0 and ϵ=0.1. Our results can be found in Figure 32. In these profiles (with the exception of the profile for $II_{D}$), we have also included numerical results carried out in *HiGFlow* using a standard LPTT model (without yield stress) for reference in order to illustrate how the solutions from the LPTT-Bingham model deviate from the standard viscoelastic model.

**Figure 33:**
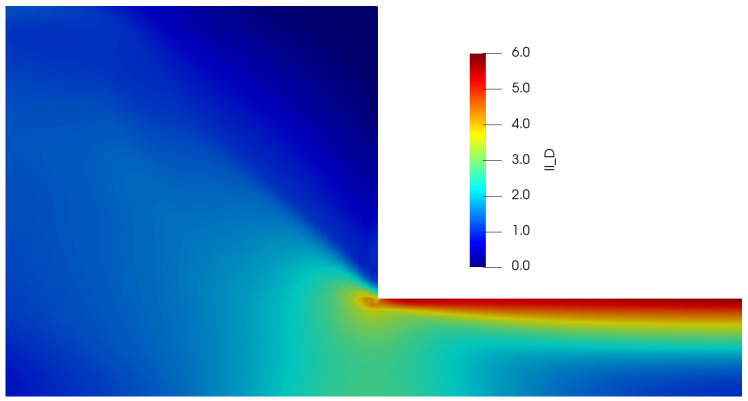
Heat map of the second invariant of the deviatoric stress tensor $\sigma_{d}$ ($II_{D}$) of a LPTT-Bingham fluid in the planar-contraction 4:1.

Firstly, it can be easily seen that the solutions obtained in both *HiGFlow* and *RheoTool* are in excellent agreement, since the curves overlap with each other. We can also observe similar behaviours in the profiles compared to the figures of the Oldroyd-B-Herschel-Bulkley (see Figure 30): an overshoot is seen for the velocity profile, while the relevant components of the elastic stress tensor become more important when the fluid enters the contraction region. More interestingly, notice how the results differ from the classic LPTT model; particularly, the stress $T_{xx}$ is much higher at the origin (x=0) for the LPTT-Bingham fluid. Similarly to the Oldroyd-B-Herschel-Bulkley case, we did not find vortexes in the corner either here, and this is because the fluid behaves as solid in the corner, see Figure 33.

### 4.5. Conclusions

In this work, we have presented numerical results from viscoelastic rheological models that have recently been implemented into the *HiGTree/HiGFlow* system, a CFD software that uses a finite difference method with meshless interpolations in hierarchical tree-based grids [25]. The implemented models (which are the Vasquez-Cook-McKinley (VCM) [8] and the Saramito model [23,24]) can describe complex rheological behaviour of fluids that are of great interest in the industry, such as micellar solutions that exhibit viscoelasticity and shear-banding and elastoviscoplastic materials.

Using experimental reported values of the fluid parameters and typical flow conditions, these models have been tested in *HiGFlow* using benchmark geometries, such as the two-dimensional channel and the 4:1 planar-contraction, where we carried out unsteady-inertial-flow simulations. Our results were directly compared with those obtained in the open-source software *RheoTool*, a tool based on *OpenFOAM* developed to simulate generalised-Newtonian and viscoelastic flows. The main difference between the *HiGTree/HiGFlow* system and *OpenFOAM/RheoTool* is that the latter discretises the governing and constitutive equations with the finite volume method technique instead of finite differences. In this work, we have found an excellent agreement between the steady-state results predicted by both methodologies. In addition, our 2D-channel simulations were also compared with a third methodology: an in-house code that uses the Vorticity-Velocity-Formulation (*VVF*), whose numerical solutions are also similar to the results predicted by *HiGFlow* and *RheoTool*. To our knowledge, we are also the first authors to carry out computational simulations of the Vasquez-Cook-McKinley (VCM) model in the planar-contraction 4:1 geometry.

In addition, we carried out a mesh convergence analysis of our models using uniform meshes and two kinds of refined meshes (with two and three refinement levels). The results clearly showed that the numerical errors obtained for the uniform meshes with very small cell sizes are rough of the same order of magnitude as the errors obtained in our refined meshes (with identical cell sizes to those of the uniform meshes). This suggests that we can opt to use refined meshes instead of uniform meshes with very small cell sizes (which can take plenty of simulation time) since we have shown great convergence results thanks to the finite difference method and the meshless interpolation scheme used by the system *HiGTree/HiGFlow* described in Section 3.1.

We have also shown that the VCM and Saramito models implemented in the system *HiGTree/HiGFlow* can reproduce rheological behaviour that is commonly observed in complex fluids, such as the non-monotonic stress vs. shear rate flow curves of shear-banded flows, the plug-flow velocity profiles seen in viscoelastic yield-stress fluids, formation of corner vortex and non-zero normal stresses in 4:1 planar-contraction flows. For future work, it would be of great interest to implement models that incorporate thixotropy into viscoelastic yield-stress fluids, such as the MK-IKH [43] and the de Souza [22] models. Another big step to extend the library of models available in *HiGflow* would be to incorporate the heat-transfer equations in order to simulate non-isothermic flows of complex fluids [44,45,46,47,48].

## Figures and Tables

**Table 1:** Uniform meshes used for the 2D channel.

Meshes	Δx=Δy
MI (8×80)	0.125
MII (16×160)	0.0625
MIII (32×320)	0.03125
MIV (64×640)	0.015625

**Table 2:** Non–uniform refined meshes with two refinement levels used in the 2D channel.

Meshes	*Large* Δx	*Small* Δx
RMI	0.125	0.0625
RMII	0.0625	0.03125
RMIII	0.03125	0.015625

**Table 3:** Non-uniform refined meshes with three refinement levels used in the 2D channel.

Meshes	*Large* Δx	*Middle* Δx	*Small* Δx
RMIV	0.125	0.0625	0.03125
RMV	0.0625	0.03125	0.015625

**Table 4:** Errors for the velocity profile in the 2D channel–flow. For the calculation of these errors, the mesh MIV was assumed as a reference solution.

Velocity $u_{x}$ Errors			
**Mesh**	$L_{1}$	$L_{2}$	$L_{\infty }$
MI	$5.087\times 10^{-2}$	$6.997\times 10^{-2}$	$1.8759\times 10^{-1}$
MII	$5.147\times 10^{-2}$	$7.023\times 10^{-2}$	$1.9014\times 10^{-1}$
MIII	$5.167\times 10^{-2}$	$7.039\times 10^{-2}$	$1.9202\times 10^{-1}$
RMI	$5.192\times 10^{-2}$	$7.073\times 10^{-2}$	$1.9032\times 10^{-1}$
RMII	$5.156\times 10^{-2}$	$7.029\times 10^{-2}$	$1.8733\times 10^{-1}$
RMIII	$3.849\times 10^{-2}$	$4.117\times 10^{-2}$	$5.5436\times 10^{-2}$
RMIV	$5.162\times 10^{-2}$	$7.032\times 10^{-2}$	$1.8404\times 10^{-1}$
RMV	$8.800\times 10^{-3}$	$1.060\times 10^{-2}$	$2.4743\times 10^{-2}$

**Table 5:** Errors for the normal stress $T_{xx}$ profile in the 2D channel–flow. For the calculation of these errors, the mesh MIV was assumed as a reference solution.

Stress $T_{\mathrm{xx}}$ Errors			
**Mesh**	$L_{1}$	$L_{2}$	$L_{\infty }$
MI	$1.541\times 10^{-1}$	$2.660\times 10^{-1}$	$6.1488\times 10^{-1}$
MII	$5.421\times 10^{-2}$	$1.722\times 10^{-1}$	$3.9556\times 10^{-1}$
MIII	$4.131\times 10^{-2}$	$1.177\times 10^{-1}$	$3.1724\times 10^{-1}$
RMI	$7.595\times 10^{-2}$	$2.670\times 10^{-1}$	$6.1718\times 10^{-1}$
RMII	$5.380\times 10^{-2}$	$1.675\times 10^{-1}$	$3.7873\times 10^{-1}$
RMIII	$2.543\times 10^{-2}$	$5.958\times 10^{-2}$	$1.0801\times 10^{-1}$
RMIV	$7.829\times 10^{-2}$	$2.668\times 10^{-1}$	$6.1063\times 10^{-1}$
RMV	$2.553\times 10^{-2}$	$1.130\times 10^{-1}$	$3.3298\times 10^{-1}$

**Table 6:** Errors for the shear stress $T_{xy}$ profile in the 2D channel–flow. For the calculation of these errors, the mesh MIV was assumed as a reference solution.

Stress $T_{\mathrm{xy}}$ Errors			
**Mesh**	$L_{1}$	$L_{2}$	$L_{\infty }$
MI	$1.448\times 10^{-1}$	$1.887\times 10^{-1}$	$3.7420\times 10^{-1}$
MII	$7.403\times 10^{-2}$	$9.697\times 10^{-2}$	$2.3815\times 10^{-1}$
MIII	$6.985\times 10^{-2}$	$7.817\times 10^{-2}$	$2.1894\times 10^{-1}$
RMI	$1.222\times 10^{-1}$	$1.631\times 10^{-1}$	$2.8733\times 10^{-1}$
RMII	$7.580\times 10^{-2}$	$9.162\times 10^{-2}$	$2.2184\times 10^{-1}$
RMIII	$9.389\times 10^{-2}$	$1.368\times 10^{-1}$	$3.1017\times 10^{-1}$
RMIV	$1.159\times 10^{-1}$	$1.422\times 10^{-1}$	$2.1485\times 10^{-1}$
RMV	$3.492\times 10^{-2}$	$4.892\times 10^{-2}$	$9.2251\times 10^{-2}$

**Table 7:** Errors for the density number $n_{A}$ of specie A profile in the 2D channel–flow. For the calculation of these errors, the mesh MIV was assumed as a reference solution.

Density Number $n_{A}$ Errors			
**Mesh**	$L_{1}$	$L_{2}$	$L_{\infty }$
MI	$2.285\times 10^{-1}$	$3.248\times 10^{-1}$	$5.2077\times 10^{-1}$
MII	$1.341\times 10^{-1}$	$1.789\times 10^{-1}$	$2.6022\times 10^{-1}$
MIII	$1.137\times 10^{-1}$	$1.495\times 10^{-1}$	$2.3416\times 10^{-1}$
RMI	$2.078\times 10^{-1}$	$2.987\times 10^{-1}$	$4.8055\times 10^{-1}$
RMII	$1.337\times 10^{-1}$	$1.771\times 10^{-1}$	$2.6180\times 10^{-1}$
RMIII	$6.575\times 10^{-2}$	$7.918\times 10^{-2}$	$1.0642\times 10^{-1}$
RMIV	$2.061\times 10^{-1}$	$2.986\times 10^{-1}$	$4.8870\times 10^{-1}$
RMV	$5.878\times 10^{-2}$	$8.438\times 10^{-2}$	$1.3690\times 10^{-1}$

**Table 8:** Errors for the density number $n_{B}$ of specie B profile in the 2D channel–flow. For the calculation of these errors, the mesh MIV was assumed as a reference solution.

Density Number $n_{B}$ Errors			
**Mesh**	$L_{1}$	$L_{2}$	$L_{\infty }$
MI	$6.507\times 10^{-2}$	$1.008\times 10^{-1}$	$2.3092\times 10^{-1}$
MII	$4.045\times 10^{-2}$	$6.104\times 10^{-2}$	$1.4938\times 10^{-1}$
MIII	$3.500\times 10^{-2}$	$5.290\times 10^{-2}$	$1.5076\times 10^{-1}$
RMI	$5.943\times 10^{-2}$	$9.385\times 10^{-2}$	$2.1689\times 10^{-1}$
RMII	$4.024\times 10^{-2}$	$6.042\times 10^{-2}$	$1.5066\times 10^{-1}$
RMIII	$2.253\times 10^{-2}$	$3.054\times 10^{-2}$	$6.2624\times 10^{-2}$
RMIV	$5.843\times 10^{-2}$	$9.323\times 10^{-2}$	$2.2011\times 10^{-1}$
RMV	$1.949\times 10^{-2}$	$3.119\times 10^{-2}$	$7.4277\times 10^{-2}$

**Table 9:** Errors for the velocity profile in the 2D channel–flow. These errors correspond to the solutions obtained by *HiGFlow* using the Oldroyd–B–Bingham model. For the calculation of these errors, the mesh MIV was assumed as a reference solution.

Velocity $u_{x}$ Errors			
**Mesh**	$L_{1}$	$L_{2}$	$L_{\infty }$
MI	$1.242\times 10^{-2}$	$1.313\times 10^{-2}$	$2.0495\times 10^{-2}$
MII	$2.030\times 10^{-3}$	$2.670\times 10^{-3}$	$5.711\times 10^{-3}$
MIII	$6.162\times 10^{-4}$	$6.780\times 10^{-4}$	$1.574\times 10^{-3}$
RMI	$4.760\times 10^{-3}$	$6.678\times 10^{-3}$	$1.435\times 10^{-2}$
RMII	$1.694\times 10^{-3}$	$2.343\times 10^{-3}$	$5.578\times 10^{-3}$
RMIII	$4.388\times 10^{-4}$	$5.357\times 10^{-4}$	$1.367\times 10^{-3}$
RMIV	$4.910\times 10^{-3}$	$6.721\times 10^{-3}$	$1.559\times 10^{-2}$
RMV	$1.910\times 10^{-3}$	$2.322\times 10^{-3}$	$4.941\times 10^{-3}$

**Table 10:** Errors for the normal stress $T_{xx}$ profile in the 2D channel–flow. These errors correspond to the solutions obtained by *HiGFlow* using the Oldroyd–B–Bingham model. For the calculation of these errors, the mesh MIV was assumed as a reference solution.

Stress $T_{\mathrm{xx}}$ Errors			
**Mesh**	$L_{1}$	$L_{2}$	$L_{\infty }$
MI	$4.620\times 10^{-2}$	$4.067\times 10^{-2}$	$3.9578\times 10^{-2}$
MII	$4.898\times 10^{-3}$	$6.592\times 10^{-3}$	$9.906\times 10^{-3}$
MIII	$2.817\times 10^{-3}$	$2.739\times 10^{-3}$	$3.306\times 10^{-3}$
RMI	$2.916\times 10^{-2}$	$3.410\times 10^{-2}$	$3.516\times 10^{-2}$
RMII	$6.696\times 10^{-3}$	$7.263\times 10^{-3}$	$9.832\times 10^{-3}$
RMIII	$1.719\times 10^{-3}$	$2.033\times 10^{-3}$	$4.331\times 10^{-3}$
RMIV	$3.329\times 10^{-2}$	$3.633\times 10^{-2}$	$3.498\times 10^{-2}$
RMV	$9.243\times 10^{-3}$	$8.818\times 10^{-3}$	$1.1062\times 10^{-2}$

**Table 11:** Errors for the shear stress $T_{xy}$ profile in the 2D channel–flow. These errors correspond to the solutions obtained by *HiGFlow* using the Oldroyd–B–Bingham model. For the calculation of these errors, the mesh MIV was assumed as a reference solution.

Stress $T_{\mathrm{xy}}$ Errors			
**Mesh**	$L_{1}$	$L_{2}$	$L_{\infty }$
MI	$3.014\times 10^{-2}$	$3.346\times 10^{-2}$	$4.785\times 10^{-2}$
MII	$1.491\times 10^{-2}$	$2.077\times 10^{-2}$	$2.957\times 10^{-2}$
MIII	$8.861\times 10^{-3}$	$1.244\times 10^{-2}$	$2.788\times 10^{-2}$
RMI	$2.645\times 10^{-2}$	$3.562\times 10^{-2}$	$5.319\times 10^{-2}$
RMII	$1.382\times 10^{-2}$	$1.865\times 10^{-2}$	$2.718\times 10^{-2}$
RMIII	$4.445\times 10^{-3}$	$7.923\times 10^{-3}$	$2.076\times 10^{-2}$
RMIV	$3.267\times 10^{-2}$	$4.105\times 10^{-2}$	$5.434\times 10^{-2}$
RMV	$1.204\times 10^{-2}$	$1.468\times 10^{-2}$	$2.210\times 10^{-2}$

**Table 12:** Errors for the second invariant of the deviatoric stress tensor $\sigma_{d}$ ($II_{D}$) profile in the 2D channel–flow. These errors correspond to the solutions obtained by *HiGFlow* using the Oldroyd–B–Bingham model. For the calculation of these errors, the mesh MIV was assumed as a reference solution.

$\sigma_{d}$ Errors			
MI	$3.791\times 10^{-2}$	$3.340\times 10^{-2}$	$3.8205\times 10^{-2}$
MII	$5.570\times 10^{-3}$	$7.056\times 10^{-3}$	$9.071\times 10^{-3}$
MIII	$4.186\times 10^{-3}$	$4.934\times 10^{-3}$	$7.517\times 10^{-3}$
RMI	$2.250\times 10^{-2}$	$2.513\times 10^{-2}$	$2.913\times 10^{-2}$
RMII	$6.675\times 10^{-3}$	$7.231\times 10^{-3}$	$8.833\times 10^{-3}$
RMIII	$2.177\times 10^{-3}$	$2.772\times 10^{-3}$	$5.446\times 10^{-3}$
RMIV	$2.720\times 10^{-2}$	$2.837\times 10^{-2}$	$2.894\times 10^{-2}$
RMV	$8.027\times 10^{-3}$	$7.828\times 10^{-3}$	$8.774\times 10^{-3}$

## Data Availability

Not applicable.

## Abbreviations

The following abbreviations are used in this manuscript:

CFD	Computational Fluid Dynamics
FVM	Finite Volume Method
MLS	Moving Least Squares
*VVF*	Vorticity-Velocity Formulation
VCM	Vasquez-Cook-McKinley
PTT	Phan–Thien-Tanner
LPTT	Linear Phan–Thien-Tanner
EPTT	Exponential Phan–Thien-Tanner
